# Genetic variants of *GADD45A, GADD45B* and *MAPK14* predict platinum-based chemotherapy-induced toxicities in Chinese patients with non-small cell lung cancer

**DOI:** 10.18632/oncotarget.8052

**Published:** 2016-03-14

**Authors:** Ming Jia, Meiling Zhu, Mengyun Wang, Menghong Sun, Ji Qian, Fei Ding, Jianhua Chang, Qingyi Wei

**Affiliations:** ^1^ Cancer Institute, Collaborative Innovation Center for Cancer Medicine, Fudan University Shanghai Cancer Center, Shanghai, 200032, China; ^2^ Department of Oncology, Shanghai Medical College, Fudan University, Shanghai, 200032, China; ^3^ Department of Oncology, Xinhua Hospital Affiliated to Shanghai Jiaotong University, School of Medicine, Shanghai, 200092, China; ^4^ Department of Pathology, Fudan University Shanghai Cancer Center, Shanghai, 200032, China; ^5^ State Key Laboratory of Genetic Engineering and MOE Key Laboratory of Contemporary Anthropology, School of Life Sciences and Fudan Taizhou Institute of Health Sciences, Fudan University, Shanghai, 200032, China; ^6^ Department of Medical Oncology, Fudan University Shanghai Cancer Center, Shanghai, 200032, China; ^7^ Department of Medicine, Duke University School of Medicine and Duke Cancer Institute, Duke University Medical Center, Durham, North Carolina, 27710, USA

**Keywords:** JNK and P3α pathways, genetic variants, lung cancer, platinum-based chemotherapy, adverse events

## Abstract

The JNK and P38α pathways play a crucial role in tissue homeostasis, apoptosis and autophagy under genotoxic stresses, but it is unclear whether single nucleotide polymorphisms (SNPs) of genes in these pathways play a role in platinum-based chemotherapy-induced toxicities in patients with advanced non-small cell lung cancer (NSCLC). We genotyped 11 selected, independent, potentially functional SNPs of nine genes in the JNK and P38α pathways in 689 patients with advanced NSCLC treated with platinum-combination chemotherapy regimens. Associations between these SNPs and chemotherapy toxicities were tested in a discovery group of 345 patients and then validated in a replication group of 344 patients. In both discovery and validation groups as well as their pooled analysis, carriers of *GADD45B* rs2024144T variant allele had a significantly higher risk for severe hematologic toxicity and carriers of *MAPK14* rs3804451A variant allele had a significantly higher risk for both overall toxicity and gastrointestinal toxicity. In addition, carriers of *GADD45A* rs581000C had a lower risk of anemia, while carriers of *GADD45B* rs2024144T had a significantly higher risk for leukocytopenia or agranulocytosis. The present study provides evidence that genetic variants in genes involved in the JNK and P38α pathways may predict platinum-based chemotherapy toxicity outcomes in patients with advanced NSCLC. Larger studies of other patient populations are needed to validate our findings.

## INTRODUCTION

Lung cancer is one of the most fatal human malignancies, the leading cause of cancer-related deaths in China [1]. Although much research effort has been made in improving treatment for lung cancer in recent decades, the five years survival rate is still less than 20% [1, 2].

Platinum (cisplatin or carboplatin) double-agent chemotherapy is recommended as the first-line treatment for patients with advanced non–small cell lung cancer (NSCLC) without sensitive *EGFR* mutations according to the National Comprehensive Cancer Network Clinical Practice Guidelines in Oncology (NCCN guidelines, http://www.nccn.org/professionals/). Platinum compounds induce DNA damage, inhibit DNA replication, and activate a number of signal transduction pathways leading to cancer cell death [3]. However, these drugs also damage normal cells, leading to severe adverse events [4, 5]. Despite initial tumor control, some cancer patients treated with chemotherapies often need to decrease drug doses or stop treatment due to severe adverse events.

The growth arrest and DNA damage-inducible 45 (*GADD45*) gene family encode three related GADD45 proteins, GADD45 α, β, and γ. These proteins are remarkably similar, but not identical, in their functions in different apoptotic and growth inhibitory pathways [6]. Previous studies have reported that DNA damage and other environmental stresses may induce the GADD45-like gene expression either through TP53 or by other means [7–9]. Once GADD45 proteins are induced, they participate in cell cycle arrest [10], DNA repair [11] or apoptosis [12]. All three GADD45 family members directly activate MTK1/MEKK4 kinases in response to environmental stresses, resulting in apoptosis induction through the JNK and P38 pathways [13], while GADD45β can also inhibit the JNK kinase MKK7 to suppress the JNK pathway signaling [14].

The JNK and P38 MAPK pathways are also called the stress activated protein kinase pathways. They control cell proliferation, differentiation, survival, migration of specific cell types and have key roles in tissue homeostasis [15, 16]. For example, the JNK and P38 pathways have been shown to be involved in apoptosis and autophagic death after genotoxic stresses and ultraviolet radiation [17]. A recent study has reported an anti-apoptotic role of the activated JNK in adipocyte-like cells treated with cisplatin [18]. These results indicate that the activation of the JNK and P38α pathways probably decrease the adverse events in NSCLC patients treated with platinum-based chemotherapies. Previous studies also reported that genetic variants in genes involved in DNA repair and apoptosis were associated with treatment-related toxicities in NSCLC patients [19–22]. Therefore, we performed a retrospective study in a patient population with advanced NSCLC to assess the role of genetic variants of genes in JNK and P38α pathways in adverse events in response to platinum-based chemotherapy.

## RESULTS

The characteristics and toxicity events in this patient population are summarized in Table 1 for both the discovery and validation groups, and all the patients had advanced NSCLC treated with platinum-based chemotherapy as the first-line treatment. As shown in Table 1, the median age at diagnosis was 58 years (range, 23-83 years). Of all the patients, 475 (68.94%) were male, 259 (37.59%) patients had TNM stage III and 430 (62.41%) had TNM stage IV diseases. The histological types included adenocarcinoma (476, 69.1%), squamous cell carcinoma (120, 17.4%), other carcinoma (64, 9.3%), and non small-cell lung cancer not otherwise specified (NSCLC-NOS) (29, 4.2%).

**Table 1:** Clinical characteristics of patients from both the discovery group and replication group in a Chinese NSCLC patient population

Patient characteristic	All patients	Discovery group	Replication group	All patients		
	N (%)	N (%)	N (%)	Overall toxicity event N (*P*^c^)	Hematologic toxicity event N (*P*^c^)	Gastrointestinal toxicity event N (*P*^c^)
All patients	689	345	344			
Median age	58	59	57			
Range	23-83	23-83	26-78			
≤58	368 (53.4)	172 (49.9)	196 (57.0)			
>58	321 (46.6)	173 (50.1)	148 (43.0)			
BMI						
≤22	29 6 (43.0)	151 (43.8)	145 (42.2)			
>22	369 (53.6)	177 (51.3)	192 (55.8)			
Missing	24 (3.5)	17 (4.9)	7 (2.04)			
Smoking status^a^						
Nonsmokers	330 (47.9)	161 (46.7)	169 (49.1)			
Ever-smokers	359 (52.1)	184 (53.3)	175 (50.9)			
Sex						
Male	475 (68.9)	244 (70.7)	231 (67.2)	167 (ref.)	146 (ref.)	31 (ref.)
Female	214 (31.1)	101 (29.3)	113 (32.9)	86 (0.205)	63 (0.732)	27 (0.009)
ECOG score						
0	192 (27.9)	84 (24.4)	108 (31.4)	76 (ref.)	67 (ref.)	13 (ref.)
1	467 (67.8)	245 (71.0)	222 (64.5)	165 (0.303)	132 (0.093)	41 (0.394)
2	30 (4.4)	16 (4.6)	14 (4.1)	12 (0.965)	10 (0.867)	4 (0.218)
TNM stage						
III	259 (37.6)	130 (37.7)	129 (37.5)	96 (ref.)	85 (ref.)	14 (ref.)
IV	430 (62.4)	215 (62.3)	215 (62.5)	157 (0.884)	124 (0.271)	44 (0.030)
Histological type						
Squamous cell carcinoma	120 (17.4)	65 (18.8)	55 (16.0)	51 (ref.)	46 (ref.)	7 (ref.)
Adenocarcinoma	476 (69.1)	237 (68.7)	239 (69.5)	164 (0.102)	131 (0.021)	44 (0.237)
Other^b^	64 (9.3)	25 (7.3)	39 (11.3)	29 (0.714)	25 (0.923)	4 (0.910)
NSCLC-NOS	29 (4.2)	18 (5.2)	11 (3.2)	9 (0.261)	7 (0.157)	3 (0.390)
Chemotherapy regimen						
Cisplatin plus pemetrexed	268 (38.9)	132 (38.3)	136 (39.5)	76 (ref.)	60 (ref.)	22 (ref.)
Carboplatin plus pemetrexed	43 (6.2)	24 (7.0)	19 (5.5)	12 (0.951)	7 (0.368)	5 (0.462)
Cisplatin plus docetaxel/paclitaxel	157 (22.8)	79 (22.9)	78 (22.7)	61 (0.026)	54 (0.007)	10 (0.489)
Carboplatin plus docetaxel/paclitaxel	85 (12.3)	42 (12.2)	43 (12.5)	47 (<.0001)	46 (<.0001)	3 (0.155)
Cisplatin plus gemcitabine	117 (17.0)	58 (16.8)	59 (17.2)	46 (0.034)	34 (0.162)	14 (0.246)
Carboplatin plus gemcitabine	19 (2.8)	10 (2.9)	9 (2.6)	11 (0.010)	8 (0.057)	4 ( 0.071)
Adverse events						
Grade 3/4 toxicity	253 (36.7)	127 (36.8)	126 (36.6)			
Grade 3/4 hematologic toxicity	209 (30.3)	102 (29.6)	107 (31.1)			
Anemia	32 (4.6)	15 (4.4)	17 (4.9)			
Leukocytopenia	94 (13.6)	48 (13.9)	46 (13.4)			
Agranulocytosis	156 (22.6)	77 (22.3)	79 (23.0)			
Thrombocytopenia	29 (4.2)	14 (4.1)	15 (4.4)			
Grade 3/4 gastrointestinal toxicity						
Nausea/vomiting/diarrhea	58 (8.4)	34 (9.9)	24 (7.0)			

a Those who had smoked < 1 cigarette daily and < 1 year in their lifetime were defined as never smokers.

b Other carcinomas include adenosquamocarcinoma, mixed cell, neuroendocrine carcinoma, and undifferentiated carcinoma.

c Obtained from unconditional logistic regression analysis.

Abbreviations: NSCLC-NOS, non small-cell lung cancer not otherwise specified

### Clinical characteristics of NSCLC patients with chemotherapy toxicities

To assess whether clinical variables that may have contributed to chemotherapy toxicities (i.e., overall toxicity, hematologic toxicity and gastrointestinal toxicity), the analysis included sex, Eastern Cooperative Oncology Group (ECOG) Performance Status Scale Score, TNM stage, histological type, and chemotherapy regimen (Table 1). We found that compared with men, more women had a risk of gastrointestinal toxicity (*P* = 0.009), and compared with TNM stage III, more stage IV patients had a risk of gastrointestinal toxicity (*P* = 0.030). Since pemetrexed was used mainly in patients with adenocarcinoma, these patients seemed to have a lower hematologic toxicity risk (*P* = 0.021).

### Association between selected SNPs and grade 3 or 4 toxicity in the discovery and replication analyses

**Figure 1:**
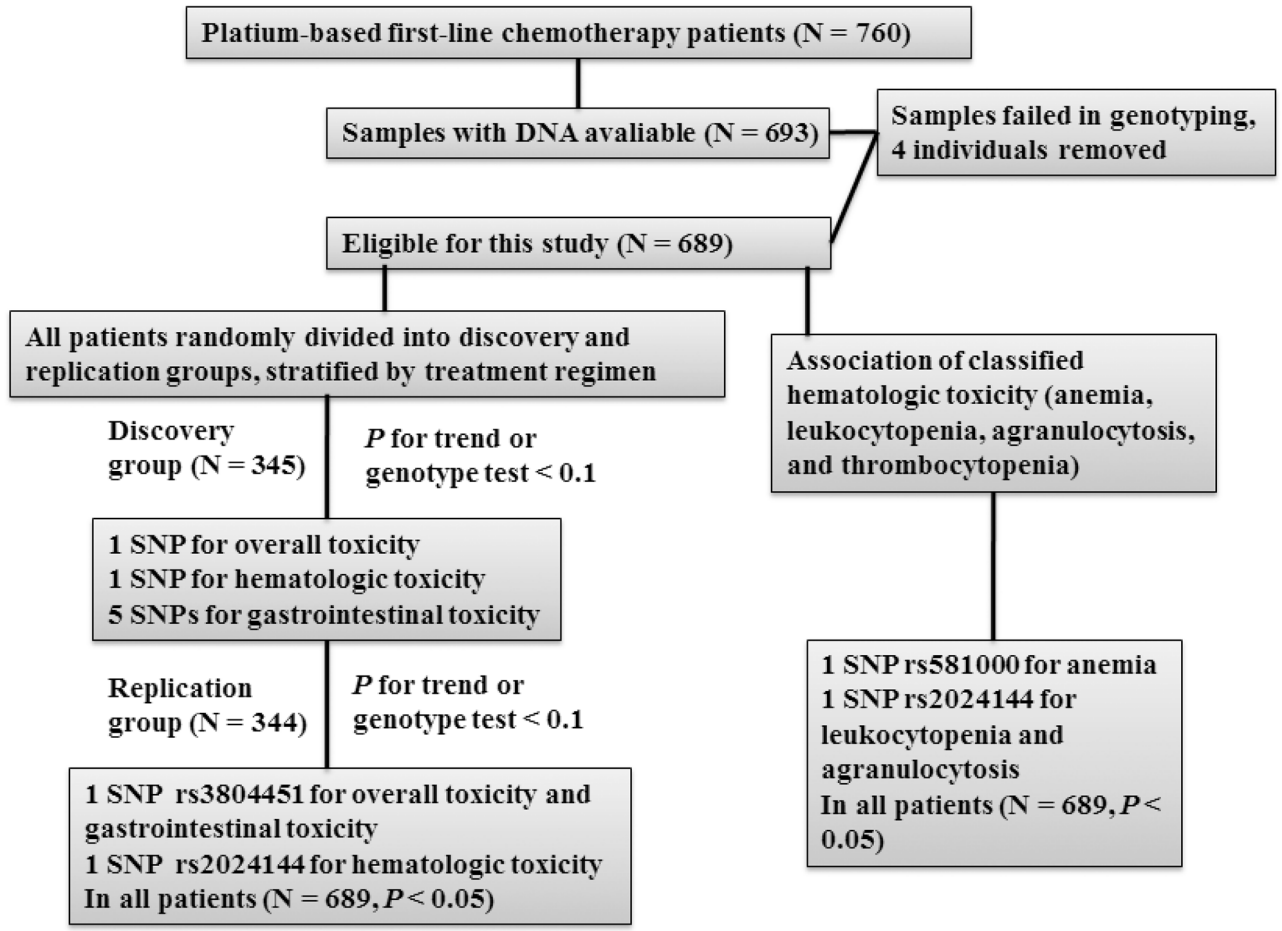
Patient recruitment strategy, including selection of eligible cases and a two-phase screening of single nucleotide polymorphisms (SNPs) associated with toxicity of platinum-based chemotherapy

In the discovery phase, we performed multivariate logistic regression analyses to estimate associations of genetic variants with toxicities and found that *MAPK14* rs3804451 SNP was associated with overall toxicity; *GADD45B* rs2024144 SNP was associated with hematologic toxicity; and *GADD45A* rs581000, *GADD45B* rs2024144, *MAP2K7* rs2115107, *MAP2K7* rs3679 and *MAPK14* rs3804451 SNPs were associated with gastrointestinal toxicity (*P* < 0.10 by the trend test or genotype test) (Figure 1, Table 2, and Supplementary Table S3–S5).

**Table 2:** Association of *MAPK14* rs3804451G>A and *GADD45B* rs2024144C>T with grade 3 or 4 chemotherapy toxicity in a Chinese NSCLC patient population

Toxicity	Genotype	Patients	Discovery group		Patients	Replication group		Patients	All patients	
		Event/N	Adjusted OR^a^ (95%CI)	*P*^a^	Event/N	Adjusted OR^a^ (95%CI)	*P*^a^	Event/N	Adjusted OR^a^ (95%CI)	*P*^a^
	*MAPK14* rs3804451			0.094^b^			0.434^b^			0.105^b^
Overall toxicity	GG	85/250	1.00 (ref.)		84/242	1.00 (ref.)		169/492	1.00 (ref.)	
	GA	39/89	1.55 (0.93-2.58)	0.092	39/90	1.65 (0.98-2.78)	0.061	78/179	**1.58 (1.10-2.27)**	**0.014**
	AA	3/6	1.60 (0.30-8.39)	0.580	3/12	0.48 (0.12-1.98)	0.311	6/18	0.73 (0.26-2.06)	0.547
	GA/AA	42/95	1.55 (0.94-2.56)	0.083	42/102	1.44 (0.87-2.37)	0.155	84/197	**1.47 (1.04-2.09)**	**0.031**
	*GADD45B* rs2024144			0.172^b^			0.131^b^			0.050^b^
Hematologic toxicity	CC	21/94	1.00 (ref.)		19/84	1.00 (ref.)		40/178	1.00 (ref.)	
	CT	59/180	**1.89 (1.04-3.44)**	**0.038**	64/191	**1.92 (1.03-3.58)**	**0.042**	123/371	**1.87 (1.22-2.87)**	**0.004**
	TT	22/71	1.59 (0.77-3.27)	0.211	24/69	1.76 (0.83-3.73)	0.142	46/140	1.62 (0.97-2.72)	0.066
	CT/TT	81/251	**1.80 (1.01-3.18)**	**0.046**	88/260	**1.87 (1.02-3.42)**	**0.042**	169/511	**1.80 (1.19-2.71)**	**0.005**
	*MAPK14* rs3804451^c^			0.056^b^			0.339^b^			0.066^b^
Gastrointestinal toxicity	GG	21/250	1.00 (ref.)		14/242	1.00 (ref.)		35/492	1.00 (ref.)	
	GA	12/89	1.94 (0.88-4.27)	0.102	10/90	2.33 (0.95-5.70)	0.064	22/179	**2.02 (1.13-3.63)**	**0.018**
	AA	1/6	4.34 (0.43-43.73)	0.213	0/12	NA	0.976	1/18	0.82 (0.10-6.96)	0.853
	GA/AA	13/95	2.02 (0.93-4.38)	0.075	10/102	1.96 (0.81-4.73)	0.133	23/197	**1.91 (1.07-3.39)**	**0.028**

a Obtained from unconditional logistic regression analysis with adjustment for sex, age at diagnosis, ECOG score and type of treatment regimen.

b *P*_trend_: *P* value for trend tests.

c Obtained from unconditional logistic regression with adjustment for sex, age at diagnosis, ECOG score, BMI, TNM stages and type of treatment regimen.

Abbreviations: CI, confidence interval; OR, odds ratio; NA, not applicable. The results were in bold, if *P* < 0.05.

The positive associations between SNPs and toxicity outcomes as defined and identified in the discovery analysis were further validated using the aforementioned criteria (*P* < 0.10 by the trend test or genotype test) in the replication (Table 1) (Figure 1, Table 2, and Supplementary Table S3–S5). Only *GADD45B* rs2024144 SNP remained to be associated with hematologic toxicity, and *MAPK14* rs3804451 SNP remained to be associated with both overall toxicity and gastrointestinal toxicity (*P* < 0.05).

### Combined analysis of all the 689 patients

We then combined the discovery and validation data to increase statistical power in further analyses of all the 689 patients (Table 2). Compared with the rs2024144CC genotype, patients with variant CT and CT/TT genotypes had a higher risk of severe hematologic toxicity (CT and CT/TT versus CC; adjusted odds ratio [OR] = 1.87 and 1.80; 95% confidence interval [CI] = 1.22-2.87 and 1.19-2.71; *P* = 0.004 and 0.005, respectively), suggesting a dominant effect of the T allele. Compared with the *MAPK14* (*P38a*) rs3804451 GG genotype, patients with the GA and GA/AA genotypes had a higher incidence of severe overall toxicity (adjusted OR =1.58 and 1.47; 95% CI = 1.10-2.27 and 1.04-2.09; *P* = 0.014 and 0.031, respectively), also suggesting a dominant effect of the A allele, which was also evident in severe gastrointestinal toxicity (adjusted OR =2.02 and 1.91; 95% CI = 1.13-3.63 and 1.07-3.39; *P* = 0.018 and 0.028 for GA and GA/AA genotypes of rs3804451, respectively) (Table 2).

**Figure 2:**
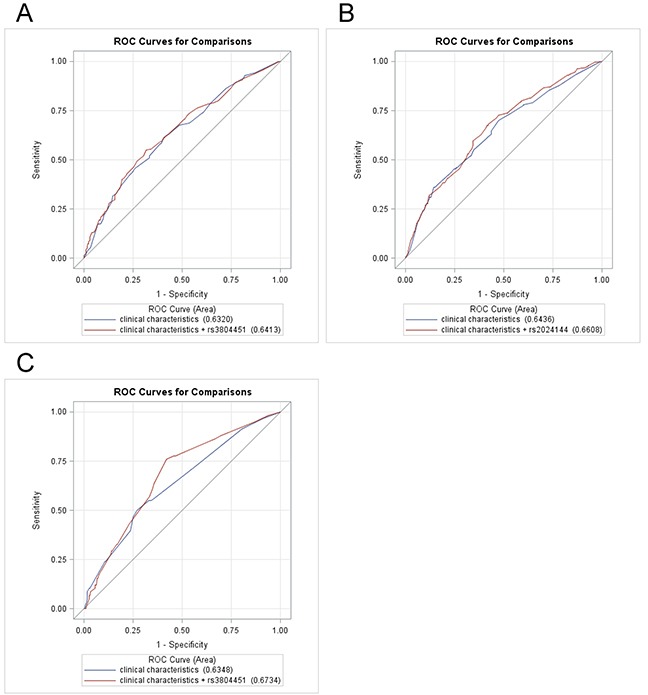
Receiver operating characteristic ROC curve with and without SNPs in prediction models of chemotherapy-induced toxicity **A.** ROC curve for prediction overall toxicity (*P* = 0.272). **B.** ROC curve for hematologic toxicity prediction (*P* = 0.121). **C.** ROC curve for gastrointestinal toxicity prediction (*P* = 0.091). A dominant model was used for all SNPs in ROC curve estimation.

We also assessed the discriminative accuracy of the prediction model with and without adding these identified SNPs to the important or significant demographic and clinical variables (age, sex, ECOG score, chemotherapy regimen, etc.) by comparing the area under the receiver operating characteristic curve (AUC) (Figure 2). When adding rs2024144 in the prediction model of hematologic toxicity, the AUC increased to 0.661 (95% CI, 0.617-0.705), compared with the model that only included age, sex, ECOG score and chemotherapy regimen (AUC, 0.644; 95% CI, 0.598-0.689; *P* = 0.121). For overall toxicity, the AUC increased slightly to 0.641 (95% CI, 0.598-0.684), when adding rs3804451 to the model compared with the AUC for the model that only included age, sex, ECOG score and chemotherapy regimen (AUC, 0.632; 95% CI, 0.589-0.675; *P* = 0.272). For gastrointestinal toxicity, the AUC increased to 0.673 (95% CI, 0.604-0.742) after including rs3804451 in the model, compared with the AUC for the model that only included age, sex, ECOG score, chemotherapy regimen, TNM stages, body mass index (BMI), and chemotherapy regimen (AUC, 0.635; 95% CI, 0.560-0.710; *P* = 0.091). These results suggest that the sample size of this study was not large enough to have a sufficient statistical power for these genetic variants to be a significant predictor in this patient study population.

### Stratified analysis of associations between selected SNPs and grade 3 or 4 toxicity

We then performed stratified analyses to evaluate the effects of variant genotypes on the hazards of chemotherapy toxicity by sex, ECOG score, TNM stage, histological type and chemotherapy regimen (Table 3). The results showed that the increased hazards of hematologic toxicity associated with the variant rs2024144T allele were more evident in subgroups who were males (*P* = 0.008), ECOG score 1 (*P* = 0.004), stage III (*P* =0.048), stage IV (*P* = 0.038) adenocarcinoma (*P* =0.034), or cisplatin plus gemcitabine (*P* = 0.030). For overall toxicity, the rs3804451 A variant allele carriers had an increased risk, particularly in males (*P* = 0.044), ECOG score 1 (*P* = 0.024), or stage IV (*P* = 0.034). For rs3804451, GG carriers also had a protective effect against gastrointestinal toxicity, particularly in patients who were males (*P* = 0.008), ECOG score 1 (*P* = 0.025), stage IV (*P* = 0.003), or cisplatin plus gemcitabine (*P* = 0.003).

**Table 3:** Stratified analysis for association of *MAPK14* rs3804451G>A and *GADD45B* rs2024144C>T with grade 3 or 4 chemotherapy toxicity in a Chinese NSCLC patient population

Variables	rs3804451 (Event/N)		Overall toxicity		rs2024144 (Event/N)		Hematologic toxicity		rs3804451 (Event/N)		Gastrointestinal toxicity	
	GG	GA/AA	Adjusted OR^b^ (95% CI)	*P*^b^	CC	CT/TT	Adjusted OR^b^ (95% CI)	*P*^b^	GG	GA/AA	Adjusted OR^c^ (95% CI)	*P*^c^
Sex												
Male	107/330	60/145	**1.54 (1.01-2.34)**	**0.044**	26/120	120/355	**1.99 (1.20-3.29)**	**0.008**	15/330	16/145	**2.83 (1.31-6.11)**	**0.008**
Female	62/162	24/52	1.62 (0.82-3.20)	0.168	14/58	49/156	1.46 (0.70-3.06)	0.315	20/162	7/52	1.32 (0.49-3.57)	0.579
ECOG score												
0	46/123	30/69	1.45 (0.77-2.73)	0.250	16/43	51/149	1.05 (0.50-2.21)	0.901	7/123	6/69	2.23 (0.60-8.31)	0.231
1	113/345	52/122	**1.66 (1.07-2.59)**	**0.024**	24/129	106/338	**2.12 (1.27-3.56)**	**0.004**	24/345	17/122	**2.19 (1.11-4.34)**	**0.025**
2	10/24	2/6	0.27 (0.02-4.85)	0.377	0/6	10/24	NA	0.944	4/24	0/6	NA	0.942
TNM stage												
III	64/179	32/80	1.27 (0.71-2.27)	0.422	15/67	70/192	**1.98 (1.01-3.89)**	**0.048**	11/179	3/80	0.68 (0.17-2.74)	0.590
IV	105/313	52/117	**1.63 (1.04-2.56)**	**0.034**	25/111	99/319	**1.76 (1.03-2.99)**	**0.038**	24/313	20/117	**2.76 (1.41-5.41)**	**0.003**
Histological type												
Squamous cell carcinoma	34/88	17/32	2.15 (0.86-5.34)	0.100	9/32	37/88	2.18 (0.82-5.74)	0.117	4/88	3/32	3.20 (0.40-25.45)	0.272
Adenocarcinoma	113/341	51/135	1.28 (0.83-1.98)	0.257	25/122	106/354	**1.74 (1.04-2.89)**	**0.034**	28/341	16/135	1.80 (0.91-3.56)	0.092
Other^b^	18/43	11/21	1.84 (0.52-6.54)	0.344	4/17	21/47	2.78 (0.64-12.15)	0.173	3/43	1/21	NA	0.917
NSCLC-NOS	4/20	5/9	4.21 (0.55-32.38)	0.167	2/7	5/22	NA	0.895	0/20	3/9	NA	0.743
Chemotherapy regimen												
Cisplatin plus pemetrexed	51/189	25/79	1.31 (0.72-2.36)	0.380	11/60	49/202	1.65 (0.79-3.45)	0.180	14/189	8/79	2.42 (0.88-6.69)	0.088
Carboplatin plus pemetrexed	11/34	1/9	0.27 (0.03-2.76)	0.269	1/10	6/33	2.49 (0.25-24.47)	0.433	4/34	1/9	NA	0.855
Cisplatin plus docetaxel/paclitaxel	41/113	20/44	1.52 (0.73-3.18)	0.265	12/44	42/113	1.71 (0.78-3.76)	0.183	7/113	3/44	1.28 (0.27-6.02)	0.754
Carboplatin plus docetaxel/paclitaxel	33/64	14/21	1.93 (0.67-5.50)	0.222	9/22	37/63	1.98 (0.73-5.34)	0.181	2/64	1/21	2.09 (0.13-34.18)	0.606
Cisplatin plus gemcitabine	28/80	18/37	2.00 (0.88-4.55)	0.098	4/30	30/87	**3.59 (1.13-11.44)**	**0.030**	5/80	9/37	**8.28 (2.09-32.79)**	**0.003**
Carboplatin plus gemcitabine	5/12	6/7	67.13 (NA)	0.069	3/6	5/13	0.71 (0.07-7.32)	0.774	3/12	1/7	0.47 (0.03-6.58)	0.578

a Those who had smoked < 1 cigarette daily and < 1 year in their lifetime were defined as never smokers.

b Adjusted for age at diagnosis, sex, ECOG score and type of treatment regimen (the stratified factor in each stratum excluded).

c Adjusted for age at diagnosis, sex, ECOG score, BMI, TNM stage and type of treatment regimen (the stratified factor in each stratum excluded).

Abbreviations: CI, confidence interval; OR, odds ratio; NA, not applicable. The results were in bold, if *P* < 0.05.

### Associations between selected SNPs and grade 3 or 4 classified hematologic toxicity

Multivariate logistic regression analyses were used to assess associations of genetic variants with leukocytopenia, agranulocytosis, anemia, and thrombocytopenia. Because of the relatively small number of cases in each subgroup, a dominant model was assumed in the genotypic association test for each SNP. For the *GADD45A* rs581000 SNP, as shown in Table 4, C allele carriers had a lower incidence of severe anemia (adjusted OR = 0.39; 95% CI = 0.18-0.82; *P* = 0.013). Increased hazards of leukocytopenia and agranulocytosis were found in the patients with the variant T allele of rs2024144 (adjusted OR = 2.08 and 1.63; 95% CI = 1.15-3.76 and 1.04-2.57; *P* = 0.015 and 0.035, respectively). No significant associations (*P* < 0.05) were found between genetic variants and risk of severe thrombocytopenia (Supplementary Table S6).

**Table 4:** Association of *GADD45A* rs581000G>C, *GADD45B* rs2024144C>T, *MPAK14* rs3804451G>A with grades 3 or 4 chemotherapy toxicity in a Chinese NSCLC patient population

All patients	rs581000 (Event/N)		Adjusted OR (95% CI)	P^*^	rs2024144 (Event/N)		Adjusted OR (95% CI)	P^*^	rs3804451 (Event/N)		Adjusted OR (95% CI)	P^*^
Toxicity	GG	GC/CC			CC	CT/TT			GG	GA/AA		
Overall toxicity^a^	97/241	156/448	0.78 (0.56-1.09)	0.142	57/178	196/511	1.37 (0.95-1.99)	0.096	169/492	84/197	**1.47** **(1.04-2.09)**	**0.031**
Hematologic toxicity^a^	78/241	131/448	0.87 (0.61-1.23)	0.418	40/178	169/511	**1.80 (1.19-2.71)**	0.005	143/492	66/197	1.24 (0.86-1.80)	0.248
Anemia^b^	17/241	15/448	**0.39 (0.18-0.82)**	**0.013**	4/178	28/511	2.61 (0.89-7.67)	0.082	24/492	8/197	0.82 (0.35-1.91)	0.639
Leukocytopenia^a^	37/241	57/448	0.80 (0.51-1.27)	0.344	15/178	79/511	**2.08 (1.15-3.76)**	0.015	62/492	32/197	1.46 (0.90-2.36)	0.123
Agranulocytosis^a^	55/241	101/448	1.01 (0.68-1.50)	0.953	31/178	125/511	**1.63 (1.04-2.57)**	**0.035**	109/492	47/197	1.12 (0.75-1.69)	0.580
Thrombocytopenia^a^	8/241	21/448	1.46 (0.63-3.41)	0.380	4/178	25/511	2.21 (0.75-6.51)	0.151	20/492	9/197	0.99 (0.43-2.24)	0.973
Gastrointestinal toxicity^c^	24/241	34/448	0.71 (0.41-1.25)	0.239	20/178	38/511	0.65 (0.36-1.16)	0.144	35/492	23/197	**1.91 (1.07-3.39)**	**0.028**

* Obtained from logistic regression analysis.

a Obtained from unconditional logistic regression with adjustment for sex, age at diagnosis, ECOG score and type of treatment regimen.

b Obtained from unconditional logistic regression with adjustment for sex, age at diagnosis, ECOG score, BMI, histological type and type of treatment regimen.

c Obtained from unconditional logistic regression with adjustment for sex, age at diagnosis, ECOG score, BMI, TNM stages and type of treatment regimen.

Abbreviations: CI, confidence interval; OR, odds ratio. The results were in bold, if *P* < 0.05.

## DISCUSSION

Platinum-based chemotherapy activates cellular signaling pathways that result in apoptosis [3, 23], unless these DNA damages are repaired before DNA replication. Molecular and cellular studies have provided ample evidence for the roles of the JNK and P38α pathways in tissue homeostasis [15, 16, 24]. It has been suggested that activation of these pathways may activate DNA repair and inhibit apoptosis in normal cells and therefore decrease the toxicity outcomes of patients receiving chemotherapies. In the present study, we found that *MAPK14* rs3804451 SNP was associated with both overall toxicity and gastrointestinal toxicity and that *GADD45B* rs2024144 SNP was associated with hematologic toxicity. All associations with these SNPs observed in both discovery and replication groups were consistently in the same direction. We also found that patients with the *GADD45A* rs581000C variant allele had a lower risk of severe anemia, whereas patients with rs2024144T variant allele had an increased risk of leukocytopenia and agranulocytosis.

Previous studies indicated that both gadd45α and gadd45β increased the survival of hematopoietic cells under conditions of certain anticancer drugs in mouse model [25]. Furthermore, it was shown that both gaddd45a and gadd45β also cooperated to promote cell survival via two distinct signaling pathways involving activation of the gadd45α-P38-NF-kB survival pathway and the gadd45β-mediated inhibition of the stress response MKK4-JNK pathway [26]. Since the *GADD45B* rs2024144T variant allele was reported to significantly repress transcription activity of *GADD45B* mRNA to inhibit the JNK signaling [27], it is not surprising that patients with the rs2024144T minor allele had a higher risk of severe hematologic toxicity in response to platinum-based chemotherapy. These previous findings and results of the present study support the notion that the rs2024144T allele may inhibit the expression of GADD45β, leading to the block of the MKK7 catalytic activity and activation of the JNK signaling to induce apoptosis of hematopoietic cells, a potential molecular mechanism underlying the risk of hematologic toxicity. In addition, the rs581000C variant allele appeared to significantly increase reporter activity of the *GADD45A* promoter [28], thus activating the GADD45α-P38-NF-kB survival pathway to increase the survival of hematopoietic cells against severe anemia. These need to be further tested in larger well-designed patient studies as well as by additional mechanistic studies.

SNP rs3804451 is located on the 3′ untranslated region (UTR) of *MAPK14* (*P38a*). Based on a bioinformatics web server (http://snpinfo.niehs.nih.gov/snpinfo/index.html), the SNP rs3804451 was predicted to bind to microRNA (miRNA) -377 at the G variant allele or bind to miRNA-485-3p and miRNA-1301 at the A allele. Therefore, the expression levels of P38a will depend on the proportions of these miRNAs or the binding affinity between miRNA and SNP rs3804451, which may lead to patients' different levels of chemotherapy toxicity in response to the platinum-based chemotherapy.

The present study also suggested that genetic variants might have an effect on overall, hematologic and gastrointestinal adverse events. For example, rs2024144 CT/TT genotypes were associated with a higher risk of severe hematologic toxicity; however they were also associated with a relatively low risk for gastrointestinal toxicity. Although the functions of the pathways are complex, individual gene or SNPs may have contribute to that complexity, which is consistent with the wide range of cellular responses that they stimulate in cancer patients [29–31]. In the present study, even in the subgroup analysis, there were some consistent associations of the SNPs with platinum-based chemotherapy toxicities. However, due to limited sample-size in subgroup analyses, these results need to be validated in larger studies.

In NSCLC, the choice of cytotoxic chemotherapy is currently based on tumor histology and patient status (age and ECOG score). However, the drug-related toxicity of chemotherapy severely affects patients' quality of life and hinders further treatment. Better predictive methods are necessary to distinguish patients who tend to experience severe toxicity during the therapy course from others who do not. It is predicted that in future personalized cancer medicine SNPs as toxicity predictive markers will be much appealing, because of the convenience of its reliable detection in a blood sample [32].

However, the present study has some limitations. First, although our sample size of 689 patients may be one of the largest studies of Chinese patients reported to date, the numbers of patients in some subgroups, such as anemia and thrombocytopenia subtypes, were still quite small. This may result in low statistical power and lead to both false-negative and false-positive findings [33]. Also, the limited number of selected functional SNPs in the nine key genes did not represent all the genes in the pathways. In addition, all patients were treated at the same hospital, not representative or generalizable to other patient populations. Studies from other hospitals patient populations are needed to validate our findings. Finally, this study was only performed in a Chinese population. Therefore, examination of these SNPs in other ethnic populations with advanced NSCLC patients is necessary to elucidate possible interethnic differences of these SNPs in chemotherapeutic adverse events.

## MATERIALS AND METHODS

### Study design and patient recruitment

In the present study, we recruited patients diagnosed with histologically confirmed advanced NSCLC from Fudan University Shanghai Cancer Center (Shanghai, China) from August 2009 and October 2013. Recruitment criteria were as follows: inoperable TNM stages III-IV tumors with the presence of a measurable or assessable lesion; no prior history of cancer except for *in situ* carcinoma; no any prior chemotherapy; ECOG Scores 0-2; chemotherapy regimens including platinum in combination with docetaxel/paclitaxel, gemcitabine or pemetrexed; no surgery treatment before chemotherapy; required laboratory values for blood tests and uronoscopy in normal range; no recent (<3 months before the date of treatment) myocardial infarction and no active congestive heart failure or cardiac arrhythmia requiring medical treatment; no uncontrolled infectious disease; no other serious medical or psychological factors that might affect adverse events; at least for two chemotherapy cycles except for those who only treated by one chemotherapy cycle and also developed severe toxicity.

Patients were considered to have developed severe toxicity, if their laboratory values met the criteria in the chemotherapy treatment period whenever the toxicity happened. The chemotherapy toxicities were assessed twice a week. Hematological toxicity and gastrointestinal toxicity were included in this study, because they are the most common short-term toxicities that affect patients' quality of life and hinder further platinum-based chemotherapy. The grade of toxicity was recorded in the medical documents of each patient using the National Cancer Institute Common Toxicity Criteria, version 3.0 [34]. Clinical data were collected from medical records of each patient [including age at diagnosis, sex, ECOG score, body mass index (BMI), smoking history, clinical TNM stages, tumor histology and the information of toxicity]. The information of toxicity included overall toxicity, gastrointestinal toxicity (nausea, vomiting and diarrhea), and hematologic toxicity (including leukocytopenia, agranulocytosis, anemia, and thrombocytopenia).

However, as a retrospective study, the information in the medical records were briefly described, so we did not have the detailed information about the grade 3 or 4 classified gastrointestinal toxicity and the exact time when toxicities occurred. This study was approved by the Institutional Review Board of Fudan University Shanghai Cancer Center. All blood samples were collected from the tissue bank of Fudan University Shanghai Cancer Center, and all participants provided a written informed consent.

### Chemotherapy regimens

All the 689 patients enrolled in the study were given the first-line platinum-based chemotherapy; that is, cisplatin (75 mg/m^2^) or carboplatin (AUC 5 mg/ml.min), administered on day 1 every 3 weeks, in combination with paclitaxel (175mg/m^2^) on day 1 every 3 weeks, docetaxel (75 mg/m^2^) on day 1 every 3 weeks, gemcitabine (1,250 mg/m^2^) on days 1 and 8 every 3 weeks, or pemetrexed (500 mg/m^2^) on day 1 every 3 weeks. All chemotherapeutic drugs were administered intravenously.

### SNP selection and genotyping

Potentially functional SNPs of nine key genes (*GADD45A, GADD45B, GADD45G, MAP2K7, MAP2K4, MAP3K4, MAPK8, MAPK9* and *MAPK14*) involved in the JNK and P38α pathways were selected from the NCBI dbSNP database (http://www.ncbi.nlm.nih.gov/projects/SNP) according to the following criteria: (1) had a minor allele frequency of at least 5% in Chinese populations, (2) in low linkage disequilibrium (LD) among each other using an r^2^ threshold of 0.8 by the Haploview software (http://www.broadinstitute.org/scientific-community/science/programs/medical-and-populationgenetics/haploview/haploview) and SNPinfo (http://snpinfo.niehs.nih.gov/), (3) at the 2 ends of these genes, such as near the 5′-end, 3′-end, 5′ UTRs, or 3′UTRs, which may be the regulatory regions of the genes, and (4) potentially affect functions as predicted by SNPinfo (http://snpinfo.niehs.nih.gov/snpinfo/snpfunc.htm) such as transcription factor binding site and miRNA binding site activity; SNPs that have been previously reported to be associated with associated with acute lung injury and inter-ventricular septum hypertrophy [27, 28] are also included.

Based on these selection criteria, 14 SNPs were identified, three of which were undetectable by the TaqMan real-time PCR method. As a result, only 11 SNPs were genotyped by the TaqMan real-time PCR method with the Sequence Detection Software on an ABI-Prism 7900 instrument according to the manufacturer's instructions (Applied Biosystems, Foster City, CA). Their predicted functions and related information are summarized in Supplementary Table S1, and those untyped SNPs that are in linkage disequilibrium (LD) with these 11 SNPs are presented in Supplementary Table S2.

Blood samples were obtained at the entry to the study from the tissue bank and stored in ethylenediaminetetraacetic acid tubes at -80°C. We extracted genomic DNA from the buffy coat fraction of the blood samples by using a blood DNA minikit (Qiagen, Inc., Valencia, CA) according to the manufacturer's instructions. The DNA purity and concentrations were determined by spectrophotometer measurement of absorbance at 260 and 280 nm. Primers and probes were supplied by Applied Biosystems with four negative controls (without DNA template) and two duplicated samples included in each 384-plate for the quality control [35]. The assays were repeated for 5% of the samples, and the results were 100% concordant. Patients' status was unrevealed in the genotyping process.

### Statistical analysis

To assess which SNPs were associated with severe chemotherapy toxicity, we compared individuals with severe toxicity (NCI-CTC grades 3–4) to those with mild or no toxicity (NCI-CTC grades 0–2). Adverse events were dichotomized by the presence or absence of (a) any grade 3 or 4 toxicity, (b) any grade 3 or 4 hematologic toxicity (anemia, leukocytopenia, agranulocytosis, and thrombocytopenia), and (c) any grade 3 or 4 gastrointestinal toxicity (nausea, vomiting and diarrhea). In order to minimize false positive report probability (FPRP) [33], a two-stage analysis was performed to investigate associations between selected SNPs and the incidence of severe chemotherapy toxicity. All subjects were assigned into two groups by random digits, i.e., odd numbers to the discovery group and even numbers to the replication group with stratification by treatment regimen as previously described [36]. As a result, the discovery group had 345 patients, while the replication group had 344 patients. The demographic and other clinical data of each group had no significant difference (data not shown). The association between each genetic variant and severe toxicity was estimated by OR and its 95% CI, using unconditional logistic regression with adjustment for age at diagnosis, sex, ECOG score, and treatment regimen. As shown in Figure 1, all SNPs were first evaluated in the discovery group, and those SNPs that had *P*-values < 0.10 by either the trend test or genotype test for associations with toxicity were validated in the replication group. Finally, SNPs that met the same criteria in the validation group were further subjected to the combined analyses for all 689 patients. The association between SNPs and classified hematologic toxicity (anemia, leukocytopenia, agranulocytosis, and thrombocytopenia) were estimated only in all patients because of small number of cases in each group. Reported *P*-values were two-sided for both groups. A *P*-value < 0.05 was defined as statistically significant in the combined analyses. Unless specified, all statistical analyses were performed using SAS software, version 9.3 (SAS Institute, Inc., Cary, NC).

## SUPPLEMENTARY TABLES











## Abbreviations

NSCLC: non–small cell lung cancer
*GADD45*: growth arrest and DNA damage-inducible 45
ECOG: Eastern Cooperative Oncology Group
BMI: body mass index
UTR: untranslated region
FPRP: false positive report probability
OR: odds ratio
CI: confidence interval
AUC: area under the receiver operating characteristic curve
ROC: operating characteristic curve
LD: linkage disequilibrium
miRNA: microRNA
