# Supplementary material for: Genetic variants of GADD45A, GADD45B and MAPK14 predict platinum-based chemotherapy-induced toxicities in Chinese patients with non-small cell lung cancer
Source: Oncotarget. 2016 Mar 14;7(18):25291–303. doi: 10.18632/oncotarget.8052 (PMC5041904; doi:10.18632/oncotarget.8052)
Supplement: Supplementary file 1 [file oncotarget-07-25291-s001.pdf]

SUPPLEMENTARY TABLES

Supplemental Table S1: Characteristics of the selected pathway genes and SNPs in the study

Genes	No. of Validated SNPs	No. of Functional SNP	Selected SNP	Chr	Position	Allelic Change	MAF* Asian/Chinese	miRNA* binding sites	TFBS*
GADD45A	26	14	rs581000#	1	67922859	G→C	C:0.363/---	---	Yes
GADD45B	17	12	rs2024144#	19	2428277	C→T	T: --- / ---	---	Yes
GADD45G	32	25	rs8252	9	91410830	C→T	T:0.071/0.061	Yes	---
MAP2K7	35	29	rs2115107	19	7874168	G→A	A: --- /0.274	---	Yes
			rs3679	19	7884430	C→T	T: 0.360/0.289	Yes	Yes
MAP2K4	257	27	rs3826392	17	11863629	T→G	G: 0.146/0.202	---	Yes
MAP3K4	325	10	rs1488	6	161458240	A→G	G: 0.289/0.304	Yes	---
			rs678290	6	161331963	T→C	C: 0.242/0.143	---	Yes
MAPK8	71	12	rs10857561	10	49278644	G→A	A: 0.292/0.337	---	Yes
MAPK9	225	15	rs6703	5	179593392	T→A	A: 0.202/0.167	---	Yes
MAPK14	44	11	rs3804451	6	36185939	G→A	G: 0.135/0.179	Yes	---

SNPs from the JNK pathway: *GADD45A* rs581000, *GADD45G* rs8252, *GADD45B* rs2024144, *MAP2K7* rs2115107, rs3679, *MAP2K4* rs3826392, *MAP3K4* rs1488, rs678290, *MAPK8* rs10857561, and *MAPK9* rs6703.

SNPs from the P38a pathway: *GADD45A* rs581000, *GADD45G* rs8252, *GADD45B* rs2024144, *MAP2K4* rs3826392, *MAP3K4* rs1488 and rs678290, and *MAPK14* rs3804451.

*Abbreviations: MAF, minor allele frequency from SNPInfo; miRNA, microRNA; TFBS, transcription factor binding sites.

#SNPs reported to be associated with acute lung injury and inter-ventricular septum hypertrophy.

Supplemental Table S2: Selected SNPs and their tagged SNPs in the same gene

Genes	Selected SNP*	Chromosome No.	SNPs in LD with the selected SNPs (r^2>=0.08) as validated in Chinese populations
GADD45A	rs581000	1	None
GADD45B	rs2024144	19	None
GADD45G	rs8252	9	rs3138499
MAP2K7	rs2115107	19	None
	rs3679	19	None
MAP2K4	rs3826392	17	rs1017743, **rs11654465*,** rs11655727, rs12051769, rs12325842, **rs1468501*,** rs1870583, rs2108496, rs2322123, rs7210799, rs757594, rs8082185
	rs678290	6	rs3729613, rs3798911, rs617514
MAP3K4	rs1488	6	rs12528950, rs3798917
MAPK8	rs10857561	10	rs10508901, rs10745266, rs10776596, rs10857560, rs11101320, rs1867584, rs1919713, rs2289805, **rs2440861*,** rs2463959, rs2698761, rs2698767, rs2889611, rs3950310, rs6537561, rs7075976, rs7086275, rs7101060, rs8428, rs9888128
MAPK9	rs6703	5	rs1127575, rs11955223, rs17627593, rs4362908, rs4481314, rs6601104, rs6896513, rs7724543, rs7726664, rs9605
MAPK14	rs3804451	6	rs16883819, rs16883860, rs16884628, rs16884660, rs16884694, rs16884919, rs2237094, rs3730327, rs3730366, **rs3761980*,** rs6457878, rs6930333, rs6934216, rs7745082, rs7745536, rs7760405, rs7761118, rs7764494, rs7770183, rs7771484, rs7775290

*Predicted to be a potentially functional SNP;

Supplementary Table S3: Association of SNPs in this study with grade 3 or 4 overall toxicity in a Chinese NSCLC patient population

See Supplementary File 1

Supplementary Table S4: Association of SNPs in this study with grade 3 or 4 hematologic toxicity in a Chinese NSCLC patient population

See Supplementary File 2

Supplementary Table S5: Association of SNPs in this study with grade 3 or 4 gastrointestinal toxicity in a Chinese NSCLC patient population

See Supplementary File 3

Supplementary Table S6: Association of SNPs in this study with grade 3 or 4 anemia, leukocytopenia, agranulocytosis and thrombocytopenia in a Chinese NSCLC patient population

See Supplementary File 4
