# Supplementary material for: Genetic variants of GADD45A, GADD45B and MAPK14 predict platinum-based chemotherapy-induced toxicities in Chinese patients with non-small cell lung cancer
Source: Oncotarget. 2016 Mar 14;7(18):25291–303. doi: 10.18632/oncotarget.8052 (PMC5041904; doi:10.18632/oncotarget.8052)
Supplement: Supplementary file 2 [file oncotarget-07-25291-s002.doc]

| **Supplemental Table S3.** Association of SNPs in this study with grade 3 or 4 overall toxicity in a Chinese NSCLC patient population | | | | | | | | | | | | | | | | | | |
| --- | --- | --- | --- | --- | --- | --- | --- | --- | --- | --- | --- | --- | --- | --- | --- | --- | --- | --- |
|  |  | **Patients** |  | | **Discovery group** |  |  | **Patients** |  | | **Replication group** | |  | **Patients** |  | | **All patients** |  |
| **Gene** | **SNP** | **Event/N** | | **Adjusted**  **OR^a^ (95% CI)** | | ***P*^a^** |  | **Event/N** | | **Adjusted**  **OR^a^ (95% CI)** | | ***P*^a^** |  | **Event/N** | | **Adjusted**  **OR^a^ (95% CI)** | | ***P*^a^** |
| *GADD45A* | rs581000 |  |  | |  | 0.881^b^ |  |  |  | |  | 0.117^b^ |  |  |  | |  | 0.223^b^ |
|  | GG | 41/105 |  | | 1.00 (reference) |  |  | 56/136 |  | | 1.00 (reference) |  |  | 97/241 |  | | 1.00 (reference) |  |
|  | GC | 64/178 |  | | 0.92（0.55-1.54） | 0.707 |  | 57/168 |  | | 0.67（0.41-1.09） | 0.107 |  | 121/346 |  | | 0.77（0.55-1.10） | 0.152 |
|  | CC | 22/62 |  | | 0.97（0.50-1.90） | 0.928 |  | 13/40 |  | | 0.64（0.29-1.39） | 0.261 |  | 35/102 |  | | 0.80（0.48-1.31） | 0.367 |
|  | GC/CC | 86/240 |  | | 0.93（0.57-1.52） | 0.777 |  | 70/208 |  | | 0.66（0.41-1.06） | 0.085 |  | 156/448 |  | | 0.78（0.56-1.09） | 0.142 |
| *GADD45G* | rs8252 |  |  | |  | 0.110^b^ |  |  |  | |  | 0.518^b^ |  |  |  | |  | 0.501^b^ |
|  | CC | 64/191 |  | | 1.00 (reference) |  |  | 68/182 |  | | 1.00 (reference) |  |  | 132/373 |  | | 1.00 (reference) |  |
|  | CT | 53/133 |  | | 1.34（0.84-2.15） | 0.226 |  | 50/135 |  | | 0.94（0.58-1.54） | 0.817 |  | 103/268 |  | | 1.13（0.81-1.58） | 0.472 |
|  | TT | 10/21 |  | | 1.86（0.72-4.78） | 0.197 |  | 8/27 |  | | 0.71（0.28-1.77） | 0.458 |  | 18/48 |  | | 1.12（0.59-2.13） | 0.731 |
|  | CT/TT | 63/154 |  | | 1.40（0.89-2.20） | 0.147 |  | 58/162 |  | | 0.90（0.56-1.44） | 0.658 |  | 121/316 |  | | 1.13（0.82-1.56） | 0.457 |
| *GADD45B* | rs2024144 |  |  | |  | 0.835^b^ |  |  |  | |  | 0.169^b^ |  |  |  | |  | 0.296^b^ |
|  | CC | 33/94 |  | | 1.00 (reference) |  |  | 24/84 |  | | 1.00 (reference) |  |  | 57/178 |  | | 1.00 (reference) |  |
|  | CT | 69/180 |  | | 1.23（0.72-2.11） | 0.448 |  | 74/191 |  | | 1.70（0.95-3.03） | 0.075 |  | 143/371 |  | | 1.42（0.96-2.10） | 0.077 |
|  | TT | 25/71 |  | | 1.04（0.54-2.03） | 0.902 |  | 28/69 |  | | 1.61（0.79-3.28） | 0.187 |  | 53/140 |  | | 1.25（0.78-2.03） | 0.357 |
|  | CT/TT | 94/251 |  | | 1.17（0.71-1.96） | 0.539 |  | 102/260 |  | | 1.67（0.96-2.93） | 0.072 |  | 196/511 |  | | 1.37（0.95-1.99） | 0.096 |
| *MAP2K7* | rs2115107 |  |  | |  | 0.740^b^ |  |  |  | |  | 0.777^b^ |  |  |  | |  | 0.974^b^ |
|  | GG | 48/135 |  | | 1.00 (reference) |  |  | 54/144 |  | | 1.00 (reference) |  |  | 102/279 |  | | 1.00 (reference) |  |
|  | GA | 64/171 |  | | 1.09（0.67-1.76） | 0.733 |  | 60/164 |  | | 1.04（0.63-1.71） | 0.879 |  | 124/335 |  | | 1.05（0.75-1.47） | 0.791 |
|  | AA | 15/39 |  | | 1.10（0.51-2.36） | 0.813 |  | 12/36 |  | | 0.82（0.36-1.84） | 0.629 |  | 27/75 |  | | 0.97（0.56-1.67） | 0.900 |
|  | GA/AA | 79/210 |  | | 1.09（0.69-1.73） | 0.717 |  | 72/200 |  | | 1.00（0.62-1.6） | 0.986 |  | 151/410 |  | | 1.03（0.74-1.43） | 0.850 |
|  | rs3679 |  |  | |  | 0.811^b^ |  |  |  | |  | 0.329^b^ |  |  |  | |  | 0.597^b^ |
|  | CC | 45/123 |  | | 1.00 (reference) |  |  | 51/131 |  | | 1.00 (reference) |  |  | 96/254 |  | | 1.00 (reference) |  |
|  | CT | 63/171 |  | | 1.03（0.63-1.70） | 0.897 |  | 60/164 |  | | 0.92（0.56-1.52） | 0.756 |  | 123/335 |  | | 0.97（0.69-1.38） | 0.881 |
|  | TT | 19/51 |  | | 1.09（0.54-2.19） | 0.810 |  | 15/49 |  | | 0.67（0.32-1.4） | 0.283 |  | 34/100 |  | | 0.86（0.52-1.42） | 0.555 |
|  | CT/TT | 82/222 |  | | 1.05（0.65-1.68） | 0.852 |  | 75/213 |  | | 0.86（0.53-1.38） | 0.533 |  | 157/435 |  | | 0.95（0.68-1.32） | 0.746 |
| *MAPK8* | rs10857561 |  |  | |  | 0.282^b^ |  |  |  | |  | 0.974^b^ |  |  |  | |  | 0.390^b^ |
|  | GG | 51/151 |  | | 1.00 (reference) |  |  | 54/154 |  | | 1.00 (reference) |  |  | 105/305 |  | | 1.00 (reference) |  |
|  | GA | 58/153 |  | | 1.15（0.7-1.87） | 0.585 |  | 65/164 |  | | 1.24（0.77-2.00） | 0.386 |  | 123/317 |  | | 1.19（0.85-1.67） | 0.312 |
|  | AA | 18/41 |  | | 1.50（0.73-3.10） | 0.274 |  | 7/26 |  | | 0.70（0.27-1.82） | 0.459 |  | 25/67 |  | | 1.15（0.65-2.02） | 0.632 |
|  | GA/AA | 76/194 |  | | 1.22（0.77-1.93） | 0.405 |  | 72/190 |  | | 1.15（0.72-1.83） | 0.560 |  | 148/384 |  | | 1.18（0.86-1.64） | 0.308 |
| *MAP2K4* | rs3826392 |  |  | |  | 0.736^b^ |  |  |  | |  | 0.821^b^ |  |  |  | |  | 0.844^b^ |
|  | TT | 81/224 |  | | 1.00 (reference) |  |  | 82/215 |  | | 1.00 (reference) |  |  | 163/439 |  | | 1.00 (reference) |  |
|  | TG | 43/107 |  | | 1.13（0.69-1.85） | 0.635 |  | 38/118 |  | | 0.88（0.53-1.44） | 0.604 |  | 81/225 |  | | 0.97（0.69-1.37） | 0.861 |
|  | GG | 3/14 |  | | 0.47（0.12-1.75） | 0.258 |  | 6/11 |  | | 2.15（0.59-7.86） | 0.249 |  | 9/25 |  | | 0.95（0.40-2.26） | 0.910 |
|  | TG/GG | 46/121 |  | | 1.03（0.64-1.66） | 0.908 |  | 44/129 |  | | 0.95（0.59-1.54） | 0.843 |  | 90/250 |  | | 0.97（0.69-1.35） | 0.847 |
| *MAPK9* | rs6703 |  |  | |  | 0.658^b^ |  |  |  | |  | 0.935^b^ |  |  |  | |  | 0.734^b^ |
|  | TT | 86/236 |  | | 1.00 (reference) |  |  | 85/227 |  | | 1.00 (reference) |  |  | 171/463 |  | | 1.00 (reference) |  |
|  | TA | 35/96 |  | | 0.96（0.58-1.61） | 0.886 |  | 36/105 |  | | 0.93（0.56-1.54） | 0.783 |  | 71/201 |  | | 0.96（0.67-1.37） | 0.803 |
|  | AA | 6/13 |  | | 1.73（0.54-5.54） | 0.359 |  | 5/12 |  | | 1.16（0.33-4.06） | 0.822 |  | 11/25 |  | | 1.49（0.64-3.48） | 0.361 |
|  | TA/AA | 41/109 |  | | 1.04（0.64-1.69） | 0.891 |  | 41/117 |  | | 0.95（0.59-1.55） | 0.845 |  | 82/226 |  | | 1.00（0.71-1.41） | 0.983 |
| *MAP3K4* | rs1488 |  |  | |  | 0.781^b^ |  |  |  | |  | 0.714^b^ |  |  |  | |  | 0.762^b^ |
|  | AA | 66/179 |  | | 1.00 (reference) |  |  | 72/194 |  | | 1.00 (reference) |  |  | 138/373 |  | | 1.00 (reference) |  |
|  | AG | 46/130 |  | | 0.89（0.54-1.46） | 0.648 |  | 49/131 |  | | 0.99（0.61-1.60） | 0.963 |  | 95/261 |  | | 0.90（0.64-1.27） | 0.555 |
|  | GG | 15/36 |  | | 1.29（0.61-2.73） | 0.509 |  | 5/19 |  | | 0.73（0.24-2.21） | 0.579 |  | 20/55 |  | | 1.02（0.56-1.87） | 0.953 |
|  | AG/GG | 61/166 |  | | 0.97（0.61-1.53） | 0.892 |  | 54/150 |  | | 0.96（0.60-1.52） | 0.848 |  | 115/316 |  | | 0.92（0.67-1.27） | 0.620 |
|  | rs678290 |  |  | |  | 0.850^b^ |  |  |  | |  | 0.859^b^ |  |  |  | |  | 0.861^b^ |
|  | TT | 95/256 |  | | 1.00 (reference) |  |  | 89/243 |  | | 1.00 (reference) |  |  | 184/499 |  | | 1.00 (reference) |  |
|  | TC | 30/79 |  | | 1.15（0.67-1.98） | 0.616 |  | 33/89 |  | | 1.07（0.63-1.81） | 0.813 |  | 63/168 |  | | 1.11（0.76-1.62） | 0.580 |
|  | CC | 2/10 |  | | 0.45（0.09-2.24） | 0.330 |  | 4/12 |  | | 0.68（0.19-2.48） | 0.561 |  | 6/22 |  | | 0.61（0.23-1.63） | 0.320 |
|  | TC/CC | 32/89 |  | | 1.05（0.62-1.78） | 0.852 |  | 37/101 |  | | 1.01（0.61-1.67） | 0.969 |  | 69/190 |  | | 1.04（0.73-1.49） | 0.831 |
| *MAPK14* | rs3804451 |  |  | |  | 0.094^b^ |  |  |  | |  | 0.434^b^ |  |  |  | |  | 0.105^b^ |
|  | GG | 85/250 |  | | 1.00 (reference) |  |  | 84/242 |  | | 1.00 (reference) |  |  | 169/492 |  | | 1.00 (reference) |  |
|  | GA | 39/89 |  | | 1.55（0.93-2.58） | 0.092 |  | 39/90 |  | | 1.65（0.98-2.78） | 0.061 |  | 78/179 |  | | **1.58（1.10-2.27）** | **0.014** |
|  | AA | 3/6 |  | | 1.60（0.30-8.40） | 0.580 |  | 3/12 |  | | 0.48（0.12-1.98） | 0.311 |  | 6/18 |  | | 0.73（0.26-2.06） | 0.547 |
|  | GA/AA | 42/95 |  | | 1.55（0.94-2.56） | 0.083 |  | 42/102 |  | | 1.44（0.87-2.37） | 0.155 |  | 84/197 |  | | **1.47（1.04-2.09）** | **0.031** |
| ^a^ Data were calculated using unconditional logistic regression, adjusted by age at diagnosis, sex, ECOG score and type of treatment regimen. | | | | | | | | | | | | | | | | | | |
| ^b^ *P*trend: *P* value for trend tests. | | |  | |  |  |  |  |  | |  |  |  |  |  | |  |  |
| Abbreviations: CI, confidence interval; OR, odds ratio. The results were in bold, if *P*<0.05. | | | | | | | | | | | | | | | | | | |
|  | | | | | |  |  |  |  | |  |  |  |  |  | |  |  |
|  |  |  |  | |  |  |  |  |  | |  |  |  |  |  | |  |  |
