# Supplementary material for: Genetic variants of GADD45A, GADD45B and MAPK14 predict platinum-based chemotherapy-induced toxicities in Chinese patients with non-small cell lung cancer
Source: Oncotarget. 2016 Mar 14;7(18):25291–303. doi: 10.18632/oncotarget.8052 (PMC5041904; doi:10.18632/oncotarget.8052)
Supplement: Supplementary file 3 [file oncotarget-07-25291-s003.doc]

| **Supplemental Table S4.** Association of SNPs in this study with grade 3 or 4 hematologic toxicity in a Chinese NSCLC patient population | | | | | | | | | | | | | | | | | | |
| --- | --- | --- | --- | --- | --- | --- | --- | --- | --- | --- | --- | --- | --- | --- | --- | --- | --- | --- |
|  |  | **Patients** |  | | **Discovery group** |  |  | **Patients** |  | | **Replication group** | |  | **Patients** |  | | **All patients** |  |
| **Gene** | **SNP** | **Event/N** | | **Adjusted**  **OR^a^ (95% CI)** | | ***P*^a^** |  | **Event/N** | | **Adjusted**  **OR^a^ (95% CI)** | | ***P*^a^** |  | **Event/N** | | **Adjusted**  **OR^a^ (95% CI)** | | ***P*^a^** |
| *GADD45A* | rs581000 |  |  | |  | 0.253^b^ |  |  |  | |  | 0.108^b^ |  |  |  | |  | 0.685^b^ |
|  | GG | 30/105 |  | | 1.00 (reference) |  |  | 48/136 |  | | 1.00 (reference) |  |  | 78/241 |  | | 1.00 (reference) |  |
|  | GC | 50/178 |  | | 1.07（0.61-1.88） | 0.803 |  | 49/168 |  | | 0.70（0.42-1.16） | 0.167 |  | 99/346 |  | | 0.84（0.58-1.21） | 0.340 |
|  | CC | 22/62 |  | | 1.55（0.77-3.13） | 0.218 |  | 10/40 |  | | 0.58（0.25-1.32） | 0.194 |  | 32/102 |  | | 0.97（0.58-1.63） | 0.914 |
|  | GC/CC | 72/240 |  | | 1.19（0.70-2.02） | 0.523 |  | 59/208 |  | | 0.67（0.41-1.10） | 0.112 |  | 131/448 |  | | 0.87（0.61-1.23） | 0.418 |
| *GADD45G* | rs8252 |  |  | |  | 0.352^b^ |  |  |  | |  | 0.631^b^ |  |  |  | |  | 0.737^b^ |
|  | CC | 54/191 |  | | 1.00 (reference) |  |  | 56/182 |  | | 1.00 (reference) |  |  | 110/373 |  | | 1.00 (reference) |  |
|  | CT | 40/133 |  | | 1.10（0.67-1.82） | 0.707 |  | 45/135 |  | | 1.08（0.65-1.80） | 0.771 |  | 85/268 |  | | 1.10（0.77-1.57） | 0.597 |
|  | TT | 8/21 |  | | 1.73（0.65-4.65） | 0.274 |  | 6/27 |  | | 0.62（0.23-1.68） | 0.346 |  | 14/48 |  | | 1.00（0.51-1.99） | 0.994 |
|  | CT/TT | 48/154 |  | | 1.17（0.72-1.89） | 0.522 |  | 51/162 |  | | 0.99（0.61-1.61） | 0.963 |  | 99/316 |  | | 1.09（0.77-1.52） | 0.636 |
| *GADD45B* | rs2024144 |  |  | |  | 0.172^b^ |  |  |  | |  | 0.131^b^ |  |  |  | |  | 0.050^b^ |
|  | CC | 21/94 |  | | 1.00 (reference) |  |  | 19/84 |  | | 1.00 (reference) |  |  | 40/178 |  | | 1.00 (reference) |  |
|  | CT | 59/180 |  | | **1.89（1.04-3.44）** | **0.038** |  | **64/191** |  | | **1.92（1.03-3.58）** | **0.042** |  | 123/371 |  | | **1.87（1.22-2.87）** | **0.004** |
|  | TT | 22/71 |  | | 1.59（0.77-3.27） | 0.211 |  | 24/69 |  | | 1.76（0.83-3.73） | 0.142 |  | 46/140 |  | | 1.62（0.97-2.72） | 0.066 |
|  | CT/TT | 81/251 |  | | **1.80（1.01-3.18）** | **0.046** |  | **88/260** |  | | **1.87（1.02-3.42）** | **0.042** |  | 169/511 |  | | **1.80（1.19-2.71）** | **0.005** |
| *MAP2K7* | rs2115107 |  |  | |  | 0.781^b^ |  |  |  | |  | 0.661^b^ |  |  |  | |  | 0.659^b^ |
|  | GG | 38/135 |  | | 1.00 (reference) |  |  | 44/144 |  | | 1.00 (reference) |  |  | 82/279 |  | | 1.00 (reference) |  |
|  | GA | 51/171 |  | | 1.07（0.64-1.79） | 0.798 |  | 52/164 |  | | 1.20（0.71-2.02） | 0.493 |  | 103/335 |  | | 1.10（0.77-1.57） | 0.613 |
|  | AA | 13/39 |  | | 1.09（0.50-2.42） | 0.825 |  | 11/36 |  | | 1.07（0.46-2.49） | 0.874 |  | 24/75 |  | | 1.09（0.61-1.93） | 0.779 |
|  | GA/AA | 64/210 |  | | 1.07（0.66-1.76） | 0.776 |  | 63/200 |  | | 1.18（0.71-1.94） | 0.526 |  | 127/410 |  | | 1.10（0.78-1.55） | 0.606 |
|  | rs3679 |  |  | |  | 0.648^b^ |  |  |  | |  | 0.905^b^ |  |  |  | |  | 0.650^b^ |
|  | CC | 39/123 |  | | 1.00 (reference) |  |  | 42/131 |  | | 1.00 (reference) |  |  | 81/254 |  | | 1.00 (reference) |  |
|  | CT | 47/171 |  | | 0.80（0.47-1.36） | 0.410 |  | 50/164 |  | | 0.97（0.57-1.64） | 0.909 |  | 97/335 |  | | 0.87（0.61-1.26） | 0.473 |
|  | TT | 16/51 |  | | 0.92（0.44-1.91） | 0.822 |  | 15/49 |  | | 0.96（0.45-2.04） | 0.922 |  | 31/100 |  | | 0.94（0.56-1.57） | 0.802 |
|  | CT/TT | 63/222 |  | | 0.83（0.50-1.36） | 0.457 |  | 65/213 |  | | 0.97（0.59-1.60） | 0.899 |  | 128/435 |  | | 0.89（0.63-1.26） | 0.503 |
| *MAPK8* | rs10857561 |  |  | |  | 0.318^b^ |  |  |  | |  | 0.843^b^ |  |  |  | |  | 0.512^b^ |
|  | GG | 39/151 |  | | 1.00 (reference) |  |  | 47/154 |  | | 1.00 (reference) |  |  | 86/305 |  | | 1.00 (reference) |  |
|  | GA | 49/153 |  | | 1.29（0.76-2.16） | 0.344 |  | 55/164 |  | | 1.21（0.73-2.00） | 0.453 |  | 104/317 |  | | 1.25（0.87-1.78） | 0.228 |
|  | AA | 14/41 |  | | 1.36（0.63-2.91） | 0.432 |  | 5/26 |  | | 0.58（0.20-1.69） | 0.318 |  | 19/67 |  | | 1.02（0.56-1.86） | 0.958 |
|  | GA/AA | 63/194 |  | | 1.30（0.80-2.12） | 0.294 |  | 60/190 |  | | 1.11（0.68-1.81） | 0.679 |  | 123/384 |  | | 1.20（0.85-1.69） | 0.292 |
| *MAP2K4* | rs3826392 |  |  | |  | 0.877^b^ |  |  |  | |  | 0.894^b^ |  |  |  | |  | 0.974^b^ |
|  | TT | 63/224 |  | | 1.00 (reference) |  |  | 71/215 |  | | 1.00 (reference) |  |  | 134/439 |  | | 1.00 (reference) |  |
|  | TG | 37/107 |  | | 1.34（0.80-2.25） | 0.267 |  | 30/118 |  | | 0.76（0.45-1.30） | 0.320 |  | 67/225 |  | | 0.98（0.68-1.41） | 0.902 |
|  | GG | 2/14 |  | | 0.42（0.09-1.99） | 0.275 |  | 6/11 |  | | 3.04（0.8-11.46） | 0.101 |  | 8/25 |  | | 1.11（0.45-2.72） | 0.822 |
|  | TG/GG | 39/121 |  | | 1.20（0.73-1.99） | 0.474 |  | 36/129 |  | | 0.88（0.53-1.45） | 0.607 |  | 75/250 |  | | 0.99（0.70-1.41） | 0.955 |
| *MAPK9* | rs6703 |  |  | |  | 0.608^b^ |  |  |  | |  | 0.932^b^ |  |  |  | |  | 0.744^b^ |
|  | TT | 69/236 |  | | 1.00 (reference) |  |  | 73/227 |  | | 1.00 (reference) |  |  | 142/463 |  | | 1.00 (reference) |  |
|  | TA | 28/96 |  | | 0.99（0.57-1.70） | 0.961 |  | 29/105 |  | | 0.85（0.50-1.45） | 0.551 |  | 57/201 |  | | 0.91（0.63-1.33） | 0.641 |
|  | AA | 5/13 |  | | 1.76（0.53-5.88） | 0.357 |  | 5/12 |  | | 1.51（0.43-5.31） | 0.525 |  | 10/25 |  | | 1.67（0.70-3.98） | 0.244 |
|  | TA/AA | 33/109 |  | | 1.06（0.63-1.78） | 0.822 |  | 34/117 |  | | 0.91（0.54-1.51） | 0.704 |  | 67/226 |  | | 0.98（0.68-1.41） | 0.916 |
| *MAP3K4* | rs1488 |  |  | |  | 0.625^b^ |  |  |  | |  | 0.653^b^ |  |  |  | |  | 0.370^b^ |
|  | AA | 56/179 |  | | 1.00 (reference) |  |  | 61/194 |  | | 1.00 (reference) |  |  | 117/373 |  | | 1.00 (reference) |  |
|  | AG | 34/130 |  | | 0.72（0.42-1.22） | 0.223 |  | 43/131 |  | | 1.06（0.64-1.74） | 0.832 |  | 77/261 |  | | 0.85（0.60-1.22） | 0.383 |
|  | GG | 12/36 |  | | 1.05（0.48-2.32） | 0.901 |  | 3/19 |  | | 0.51（0.14-1.90） | 0.319 |  | 15/55 |  | | 0.83（0.43-1.60） | 0.577 |
|  | AG/GG | 46/166 |  | | 0.79（0.48-1.28） | 0.335 |  | 46/150 |  | | 0.98（0.61-1.60） | 0.946 |  | 92/316 |  | | 0.85（0.60-1.19） | 0.344 |
|  | rs678290 |  |  | |  | 0.775^b^ |  |  |  | |  | 0.843^b^ |  |  |  | |  | 0.775^b^ |
|  | TT | 76/256 |  | | 1.00 (reference) |  |  | 76/243 |  | | 1.00 (reference) |  |  | 152/499 |  | | 1.00 (reference) |  |
|  | TC | 26/79 |  | | 1.32（0.75-2.32） | 0.337 |  | 28/89 |  | | 1.12（0.65-1.95） | 0.680 |  | 54/168 |  | | 1.21（0.82-1.79） | 0.332 |
|  | CC | 0/10 |  | | NA | 0.975 |  | 3/12 |  | | 0.55（0.13-2.29） | 0.415 |  | 3/22 |  | | 0.34（0.09-1.20） | 0.094 |
|  | TC/CC | 26/89 |  | | 1.12（0.64-1.95） | 0.691 |  | 31/101 |  | | 1.03（0.61-1.75） | 0.903 |  | 57/190 |  | | 1.08（0.74-1.57） | 0.700 |
| *MAPK14* | rs3804451 |  |  | |  | 0.512^b^ |  |  |  | |  | 0.665^b^ |  |  |  | |  | 0.470^b^ |
|  | GG | 70/250 |  | | 1.00 (reference) |  |  | 73/242 |  | | 1.00 (reference) |  |  | 143/492 |  | | 1.00 (reference) |  |
|  | GA | 30/89 |  | | 1.28（0.75-2.19） | 0.364 |  | 31/90 |  | | 1.38（0.80-2.38） | 0.248 |  | 61/179 |  | | 1.32（0.91-1.94） | 0.148 |
|  | AA | 2/6 |  | | 0.83（0.14-4.85） | 0.834 |  | 3/12 |  | | 0.60（0.14-2.50） | 0.482 |  | 5/18 |  | | 0.66（0.22-1.99） | 0.462 |
|  | GA/AA | 32/95 |  | | 1.25（0.74-2.10） | 0.412 |  | 34/102 |  | | 1.26（0.74-2.12） | 0.394 |  | 66/197 |  | | 1.24（0.86-1.80） | 0.248 |
| ^a^ Data were calculated using unconditional logistic regression, adjusted by age at diagnosis, sex, ECOG score and type of treatment regimenmen. | | | | | | | | | | | | | | | | | | |
| ^b^ *P*trend: *P* value for trend tests. | | | | | | | | | | | | | | | | | | |
| Abbreviations: CI, confidence interval; OR, odds ratio;NA, not applicable. The results were in bold, if *P*<0.05. | | | | | | | | | | | | | | | | | | |
|  | | | | | |  |  |  |  | |  |  |  |  |  | |  |  |
|  |  |  |  | |  |  |  |  |  | |  |  |  |  |  | |  |  |
