# Supplementary material for: Genetic variants of GADD45A, GADD45B and MAPK14 predict platinum-based chemotherapy-induced toxicities in Chinese patients with non-small cell lung cancer
Source: Oncotarget. 2016 Mar 14;7(18):25291–303. doi: 10.18632/oncotarget.8052 (PMC5041904; doi:10.18632/oncotarget.8052)
Supplement: Supplementary file 4 [file oncotarget-07-25291-s004.doc]

| **Supplemental Table S5.** Association of SNPs in this study with grade 3 or 4 gastrointestinal toxicity in a Chinese NSCLC patient population | | | | | | | | | | | | | | | | | | |
| --- | --- | --- | --- | --- | --- | --- | --- | --- | --- | --- | --- | --- | --- | --- | --- | --- | --- | --- |
|  |  | **Patients** |  | | **Discovery group** |  |  | **Patients** |  | | **Replication group** | |  | **Patients** |  | | **All patients** |  |
| **Gene** | **SNP** | **Event/N** | | **Adjusted**  **OR^a^ (95% CI)** | | ***P*^a^** |  | **Event/N** | | **Adjusted**  **OR^a^ (95% CI)** | | ***P*^a^** |  | **Event/N** | | **Adjusted**  **OR^a^ (95% CI)** | | ***P*^a^** |
| *GADD45A* | rs581000 |  |  | |  | 0.105^b^ |  |  |  | |  | 0.558^b^ |  |  |  | |  | 0.150^b^ |
|  | GG | 13/105 |  | | 1.00 (reference) |  |  | 11/136 |  | | 1.00 (reference) |  |  | 24/241 |  | | 1.00 (reference) |  |
|  | GC | 19/178 |  | | 0.81（0.37-1.77） | 0.594 |  | 10/168 |  | | 0.65（0.26-1.63） | 0.361 |  | 29/346 |  | | 0.77（0.43-1.38） | 0.383 |
|  | CC | 2/62 |  | | 0.26（0.06-1.24） | 0.091 |  | 3/40 |  | | 0.85（0.21-3.36） | 0.811 |  | 5/102 |  | | 0.50（0.18-1.36） | 0.172 |
|  | GC/CC | 21/240 |  | | 0.68（0.31-1.46） | 0.317 |  | 13/208 |  | | 0.69（0.29-1.63） | 0.395 |  | 34/448 |  | | 0.71（0.41-1.25） | 0.239 |
| *GADD45G* | rs8252 |  |  | |  | 0.168^b^ |  |  |  | |  | 0.594^b^ |  |  |  | |  | 0.562^b^ |
|  | CC | 15/191 |  | | 1.00 (reference) |  |  | 15/182 |  | | 1.00 (reference) |  |  | 30/373 |  | | 1.00 (reference) |  |
|  | CT | 16/133 |  | | 1.74（0.80-3.76） | 0.161 |  | 7/135 |  | | 0.65（0.25-1.71） | 0.386 |  | 23/268 |  | | 1.11（0.62-1.98） | 0.729 |
|  | TT | 3/21 |  | | 1.82（0.45-7.38） | 0.404 |  | 2/27 |  | | 0.97（0.20-4.74） | 0.973 |  | 5/48 |  | | 1.34（0.48-3.74） | 0.577 |
|  | CT/TT | 19/154 |  | | 1.75（0.83-3.69） | 0.143 |  | 9/162 |  | | 0.71（0.29-1.72） | 0.443 |  | 28/316 |  | | 1.14（0.66-1.98） | 0.635 |
| *GADD45B* | rs2024144 |  |  | |  | 0.072^b^ |  |  |  | |  | 0.944^b^ |  |  |  | |  | 0.167^b^ |
|  | CC | 15/94 |  | | 1.00 (reference) |  |  | 5/84 |  | | 1.00 (reference) |  |  | 20/178 |  | | 1.00 (reference) |  |
|  | CT | 14/180 |  | | **0.43（0.19-0.97）** | **0.041** |  | 15/191 |  | | 1.33（0.45-3.88） | 0.605 |  | 29/371 |  | | 0.67（0.36-1.23） | 0.198 |
|  | TT | 5/71 |  | | 0.45（0.15-1.35） | 0.154 |  | 4/69 |  | | 1.01（0.25-4.08） | 0.986 |  | 9/140 |  | | 0.59（0.26-1.36） | 0.215 |
|  | CT/TT | 19/251 |  | | **0.44（0.21-0.92）** | **0.030** |  | 19/260 |  | | 1.25（0.44-3.55） | 0.678 |  | 38/511 |  | | 0.65（0.36-1.16） | 0.144 |
| *MAP2K7* | rs2115107 |  |  | |  | 0.114^b^ |  |  |  | |  | 0.092^b^ |  |  |  | |  | 0.828^b^ |
|  | GG | 11/135 |  | | 1.00 (reference) |  |  | 13/144 |  | | 1.00 (reference) |  |  | 24/279 |  | | 1.00 (reference) |  |
|  | GA | 17/171 |  | | 1.30（0.57-2.95） | 0.538 |  | 10/164 |  | | 0.58（0.24-1.41） | 0.230 |  | 27/335 |  | | 0.93（0.52-1.68） | 0.811 |
|  | AA | 6/39 |  | | 2.79（0.90-8.71） | 0.077 |  | 1/36 |  | | 0.24（0.03-1.94） | 0.180 |  | 7/75 |  | | 1.24（0.50-3.08） | 0.646 |
|  | GA/AA | 23/210 |  | | 1.51（0.69-3.29） | 0.304 |  | 11/200 |  | | 0.51（0.21-1.22） | 0.131 |  | 34/410 |  | | 0.98（0.56-1.72） | 0.946 |
|  | rs3679 |  |  | |  | 0.055^b^ |  |  |  | |  | 0.062^b^ |  |  |  | |  | 0.758^b^ |
|  | CC | 8/123 |  | | 1.00 (reference) |  |  | 11/131 |  | | 1.00 (reference) |  |  | 19/254 |  | | 1.00 (reference) |  |
|  | CT | 20/171 |  | | 2.21（0.90-5.43） | 0.084 |  | 13/164 |  | | 0.83（0.35-1.98） | 0.669 |  | 33/335 |  | | 1.40（0.77-2.56） | 0.274 |
|  | TT | 6/51 |  | | 2.82（0.87-9.18） | 0.085 |  | 0/49 |  | | NA | 0.952 |  | 6/100 |  | | 0.92（0.35-2.41） | 0.860 |
|  | CT/TT | 26/222 |  | | 2.32（0.97-5.54） | 0.058 |  | 13/213 |  | | 0.62（0.26-1.47） | 0.279 |  | 39/435 |  | | 1.29（0.72-2.32） | 0.387 |
| *MAPK8* | rs10857561 |  |  | |  | 0.622^b^ |  |  |  | |  | 0.527^b^ |  |  |  | |  | 0.949^b^ |
|  | GG | 17/151 |  | | 1.00 (reference) |  |  | 8/154 |  | | 1.00 (reference) |  |  | 25/305 |  | | 1.00 (reference) |  |
|  | GA | 13/153 |  | | 0.71（0.32-1.56） | 0.389 |  | 14/164 |  | | 1.52（0.60-3.84） | 0.373 |  | 27/317 |  | | 0.99（0.55-1.77） | 0.966 |
|  | AA | 4/41 |  | | 0.93（0.28-3.08） | 0.908 |  | 2/26 |  | | 1.21（0.23-6.29） | 0.822 |  | 6/67 |  | | 1.06（0.41-2.76） | 0.905 |
|  | GA/AA | 17/194 |  | | 0.75（0.36-1.57） | 0.445 |  | 16/190 |  | | 1.47（0.60-3.63） | 0.399 |  | 33/384 |  | | 1.00（0.57-1.74） | 1.000 |
| *MAP2K4* | rs3826392 |  |  | |  | 0.773^b^ |  |  |  | |  | 0.443^b^ |  |  |  | |  | 0.792^b^ |
|  | TT | 22/224 |  | | 1.00 (reference) |  |  | 13/215 |  | | 1.00 (reference) |  |  | 35/439 |  | | 1.00 (reference) |  |
|  | TG | 11/107 |  | | 0.96（0.44-2.13） | 0.926 |  | 11/118 |  | | 2.09（0.85-5.15） | 0.109 |  | 22/225 |  | | 1.32（0.74-2.35） | 0.343 |
|  | GG | 1/14 |  | | 0.67（0.08-5.62） | 0.708 |  | 0/11 |  | | NA | 0.977 |  | 1/25 |  | | 0.44（0.06-3.42） | 0.430 |
|  | TG/GG | 12/121 |  | | 0.93（0.43-2.00） | 0.850 |  | 11/129 |  | | 1.79（0.74-4.35） | 0.197 |  | 23/250 |  | | 1.22（0.69-2.14） | 0.500 |
| *MAPK9* | rs6703 |  |  | |  | 0.886^b^ |  |  |  | |  | 0.944^b^ |  |  |  | |  | 0.961^b^ |
|  | TT | 23/236 |  | | 1.00 (reference) |  |  | 15/227 |  | | 1.00 (reference) |  |  | 38/463 |  | | 1.00 (reference) |  |
|  | TA | 10/96 |  | | 0.98（0.44-2.22） | 0.964 |  | 9/105 |  | | 1.39（0.57-3.39） | 0.465 |  | 19/201 |  | | 1.17（0.65-2.12） | 0.597 |
|  | AA | 1/13 |  | | 0.82（0.10-6.80） | 0.851 |  | 0/12 |  | | NA | 0.977 |  | 1/25 |  | | 0.52（0.07-3.98） | 0.525 |
|  | TA/AA | 11/109 |  | | 0.96（0.44-2.11） | 0.925 |  | 9/117 |  | | 1.23（0.51-2.98） | 0.645 |  | 20/226 |  | | 1.10（0.62-1.97） | 0.744 |
| *MAP3K4* | rs1488 |  |  | |  | 0.129^b^ |  |  |  | |  | 0.984^b^ |  |  |  | |  | 0.270^b^ |
|  | AA | 15/179 |  | | 1.00 (reference) |  |  | 14/194 |  | | 1.00 (reference) |  |  | 29/373 |  | | 1.00 (reference) |  |
|  | AG | 14/130 |  | | 1.40（0.62-3.16） | 0.413 |  | 8/131 |  | | 0.78（0.31-1.96） | 0.594 |  | 22/261 |  | | 1.02（0.56-1.85） | 0.944 |
|  | GG | 5/36 |  | | 2.42（0.78-7.51） | 0.125 |  | 2/19 |  | | 1.66（0.32-8.55） | 0.542 |  | 7/55 |  | | 2.04（0.83-5.05） | 0.123 |
|  | AG/GG | 19/166 |  | | 1.59（0.75-3.38） | 0.228 |  | 10/150 |  | | 0.87（0.37-2.07） | 0.756 |  | 29/316 |  | | 1.17（0.67-2.03） | 0.579 |
|  | rs678290 |  |  | |  | 0.647^b^ |  |  |  | |  | 0.458^b^ |  |  |  | |  | 0.409^b^ |
|  | TT | 27/256 |  | | 1.00 (reference) |  |  | 18/243 |  | | 1.00 (reference) |  |  | 45/499 |  | | 1.00 (reference) |  |
|  | TC | 5/79 |  | | 0.56（0.20-1.54） | 0.259 |  | 5/89 |  | | 0.61（0.21-1.74） | 0.353 |  | 10/168 |  | | 0.58（0.28-1.20） | 0.145 |
|  | CC | 2/10 |  | | 1.59（0.29-8.74） | 0.592 |  | 1/12 |  | | 0.89（0.10-8.12） | 0.915 |  | 3/22 |  | | 1.29（0.34-4.84） | 0.706 |
|  | TC/CC | 7/89 |  | | 0.67（0.27-1.67） | 0.394 |  | 6/101 |  | | 0.64（0.24-1.72） | 0.375 |  | 13/190 |  | | 0.66（0.34-1.28） | 0.229 |
| *MAPK14* | rs3804451 |  |  | |  | 0.056^b^ |  |  |  | |  | 0.339^b^ |  |  |  | |  | 0.066^b^ |
|  | GG | 21/250 |  | | 1.00 (reference) |  |  | 14/242 |  | | 1.00 (reference) |  |  | 35/492 |  | | 1.00 (reference) |  |
|  | GA | 12/89 |  | | 1.94（0.88-4.27） | 0.102 |  | 10/90 |  | | 2.33（0.95-5.7） | 0.064 |  | 22/179 |  | | **2.02（1.13-3.63）** | **0.018** |
|  | AA | 1/6 |  | | 4.34（0.43-43.73） | 0.213 |  | 0/12 |  | | NA | 0.976 |  | 1/18 |  | | 0.82（0.10-6.96） | 0.853 |
|  | GA/AA | 13/95 |  | | 2.02（0.93-4.38） | 0.075 |  | 10/102 |  | | 1.96（0.81-4.73） | 0.133 |  | 23/197 |  | | **1.91（1.07-3.39）** | **0.028** |
| ^a^ Data were calculated using unconditional logistic regression, adjusted by age at diagnosis, sex, ECOG score, BMI, TNM stages and type of treatment regimen. | | | | | | | | | | | | | | | | | | |
| ^b^ *P*trend: *P* value for trend tests. | | | | | | | | | | | | | | | | | | |
| Abbreviations: CI, confidence interval; OR, odds ratio; NA, not applicable. The results were in bold, if *P*<0.05. | | | | | | | | | | | | | | | | | | |
|  | | | | | | | | | | | | | | | | | | |
|  |  |  |  | |  |  |  |  |  | |  |  |  |  |  | |  |  |
