# Supplementary material for: Genetic variants of GADD45A, GADD45B and MAPK14 predict platinum-based chemotherapy-induced toxicities in Chinese patients with non-small cell lung cancer
Source: Oncotarget. 2016 Mar 14;7(18):25291–303. doi: 10.18632/oncotarget.8052 (PMC5041904; doi:10.18632/oncotarget.8052)
Supplement: Supplementary file 5 [file oncotarget-07-25291-s005.doc]

| **Supplemental Table S6.** Association of SNPs in this study with grade 3 or 4 anemia, leukocytopenia, agranulocytosis and thrombocytopenia in a Chinese NSCLC patient population.. | | | | | | | | | | | | | | | | | | | | | | | |
| --- | --- | --- | --- | --- | --- | --- | --- | --- | --- | --- | --- | --- | --- | --- | --- | --- | --- | --- | --- | --- | --- | --- | --- |
|  |  | **Patients** |  | **Anemia** |  |  | **Patients** |  | | **Leukocytopenia** | |  | **Patients** |  | | **Agranulocytosis** | |  | **Patients** |  | | **Thrombocytopenia** | |
| **Gene** | **SNP** | **Event/N** | | **Adjusted**  **OR^a^ (95% CI)** | ***P*^a^** |  | **Event/N** | | **Adjusted**  **OR^b^ (95% CI)** | | ***P*^b^** |  | **Event/N** | | **Adjusted**  **OR^b^ (95% CI)** | | ***P*^b^** |  | **Event/N** | | **Adjusted**  **OR^b^ (95% CI)** | | ***P*^b^** |
| *GADD45A* | rs581000 |  |  |  | **0.015^c^** |  |  |  | |  | 0.605^c^ |  |  |  | |  | 0.770^c^ |  |  |  | |  | 0.829^c^ |
|  | GG | 17/241 |  | 1.00 (reference) |  |  | 37/241 |  | | 1.00 (reference) |  |  | 55/241 |  | | 1.00 (reference) |  |  | 8/241 |  | | 1.00 (reference) |  |
|  | GC | 13/346 |  | **0.44 (0.21-0.95)** | **0.036** |  | 42/346 |  | | 0.77 (0.47-1.25) | 0.284 |  | 75/346 |  | | 0.94 (0.62-1.41) | 0.761 |  | 18/346 |  | | 1.60 (0.68-3.77) | 0.279 |
|  | CC | 2/102 |  | 0.25 (0.06-1.13) | 0.072 |  | 15/102 |  | | 0.93 (0.48-1.81) | 0.841 |  | 26/102 |  | | 1.14 (0.66-2.00) | 0.637 |  | 3/102 |  | | 0.87 (0.23-3.37) | 0.841 |
|  | GC/CC | 15/448 |  | **0.40 (0.19-0.84)** | **0.015** |  | 57/448 |  | | 0.81 (0.51-1.27) | 0.351 |  | 101/448 |  | | 0.98 (0.67-1.45) | 0.934 |  | 21/448 |  | | 1.43 (0.62-3.30) | 0.400 |
| *GADD45G* | rs8252 |  |  |  | 0.566^c^ |  |  |  | |  | 0.768^c^ |  |  |  | |  | 0.557^c^ |  |  |  | |  | 0.549^c^ |
|  | CC | 19/373 |  | 1.00 (reference) |  |  | 51/373 |  | | 1.00 (reference) |  |  | 79/373 |  | | 1.00 (reference) |  |  | 17/373 |  | | 1.00 (reference) |  |
|  | CT | 11/268 |  | 0.82 (0.38-1.78) | 0.622 |  | 37/268 |  | | 0.94 (0.59-1.50) | 0.806 |  | 66/268 |  | | 1.13 (0.77-1.67) | 0.525 |  | 11/268 |  | | 0.94 (0.43-2.04) | 0.865 |
|  | TT | 2/48 |  | 0.74 (0.16-3.34) | 0.691 |  | 6/48 |  | | 0.90 (0.36-2.27) | 0.830 |  | 11/48 |  | | 1.12 (0.53-2.35) | 0.767 |  | 1/48 |  | | 0.47 (0.06-3.61) | 0.466 |
|  | CT/TT | 13/316 |  | 0.81 (0.39-1.68) | 0.571 |  | 43/316 |  | | 0.94 (0.60-1.46) | 0.777 |  | 77/316 |  | | 1.13 (0.78-1.64) | 0.514 |  | 12/316 |  | | 0.86 (0.40-1.84) | 0.704 |
| *GADD45B* | rs2024144 |  |  |  | 0.332^c^ |  |  |  | |  | **0.035^c^** |  |  |  | |  | 0.070^c^ |  |  |  | |  | 0.521^c^ |
|  | CC | 4/178 |  | 1.00 (reference) |  |  | 15/178 |  | | 1.00 (reference) |  |  | 31/178 |  | | 1.00 (reference) |  |  | 4/178 |  | | 1.00 (reference) |  |
|  | CT | 23/371 |  | 2.76 (0.93-8.18) | 0.067 |  | 56/371 |  | | **2.09 (1.14-3.85)** | **0.018** |  | 87/371 |  | | **1.62 (1.01-2.59)** | **0.045** |  | 20/371 |  | | 2.38 (0.80-7.10) | 0.120 |
|  | TT | 5/140 |  | 1.83 (0.48-7.08) | 0.379 |  | 23/140 |  | | **2.13 (1.05-4.30)** | **0.036** |  | 38/140 |  | | 1.65 (0.95-2.88) | 0.077 |  | 5/140 |  | | 1.53 (0.40-5.84) | 0.535 |
|  | CT/TT | 28/511 |  | 2.54 (0.87-7.40) | 0.089 |  | 79/511 |  | | **2.10 (1.17-3.78)** | **0.013** |  | 125/511 |  | | **1.63 (1.04-2.56)** | **0.034** |  | 25/511 |  | | 2.14 (0.73-6.26) | 0.164 |
| *MAP2K7* | rs2115107 |  |  |  | 0.664^c^ |  |  |  | |  | 0.505^c^ |  |  |  | |  | 0.683^c^ |  |  |  | |  | 0.540^c^ |
|  | GG | 11/279 |  | 1.00 (reference) |  |  | 41/279 |  | | 1.00 (reference) |  |  | 61/279 |  | | 1.00 (reference) |  |  | 12/279 |  | | 1.00 (reference) |  |
|  | GA | 18/335 |  | 1.30 (0.60-2.83) | 0.502 |  | 43/335 |  | | 0.84 (0.52-1.34) | 0.451 |  | 75/335 |  | | 1.03 (0.70-1.53) | 0.875 |  | 11/335 |  | | 0.76 (0.33-1.75) | 0.521 |
|  | AA | 3/75 |  | 1.01 (0.29-4.15) | 0.889 |  | 10/75 |  | | 0.85 (0.40-1.82) | 0.678 |  | 20/75 |  | | 1.15 (0.63-2.11) | 0.652 |  | 6/75 |  | | 1.78 (0.63-5.04) | 0.278 |
|  | GA/AA | 21/410 |  | 1.27 (0.60-2.70) | 0.534 |  | 53/410 |  | | 0.84 (0.54-1.31) | 0.439 |  | 95/410 |  | | 1.05 (0.72-1.54) | 0.784 |  | 17/410 |  | | 0.95 (0.44-2.02) | 0.888 |
|  | rs3679 |  |  |  | 0.999^c^ |  |  |  | |  | 0.749^c^ |  |  |  | |  | 0.322^c^ |  |  |  | |  | 0.734^c^ |
|  | CC | 11/254 |  | 1.00 (reference) |  |  | 37/254 |  | | 1.00 (reference) |  |  | 63/254 |  | | 1.00 (reference) |  |  | 10/254 |  | | 1.00 (reference) |  |
|  | CT | 17/335 |  | 1.07 (0.49-2.35) | 0.872 |  | 42/335 |  | | 0.81 (0.50-1.32) | 0.397 |  | 70/335 |  | | 0.80 (0.53-1.19) | 0.267 |  | 14/335 |  | | 1.08 (0.47-2.47) | 0.865 |
|  | TT | 4/100 |  | 0.95 (0.29-3.12) | 0.938 |  | 15/100 |  | | 0.99 (0.51-1.92) | 0.970 |  | 23/100 |  | | 0.81 (0.46-1.42) | 0.455 |  | 5/100 |  | | 1.22 (0.40-3.70) | 0.728 |
|  | CT/TT | 21/435 |  | 1.04 (0.49-2.23) | 0.913 |  | 57/435 |  | | 0.85 (0.54-1.34) | 0.487 |  | 93/435 |  | | 0.80 (0.55-1.17) | 0.245 |  | 19/435 |  | | 1.11 (0.51-2.43) | 0.797 |
| *MAPK8* | rs10857561 |  |  |  | 0.583^c^ |  |  |  | |  | 0.816^c^ |  |  |  | |  | 0.788^c^ |  |  |  | |  | 0.957^c^ |
|  | GG | 13/305 |  | 1.00 (reference) |  |  | 38/305 |  | | 1.00 (reference) |  |  | 66/305 |  | | 1.00 (reference) |  |  | 12/305 |  | | 1.00 (reference) |  |
|  | GA | 15/317 |  | 1.13 (0.52-2.45) | 0.751 |  | 48/317 |  | | 1.15 (0.72-1.84) | 0.552 |  | 75/317 |  | | 1.03 (0.70-1.52) | 0.879 |  | 15/317 |  | | 1.29 (0.59-2.81) | 0.525 |
|  | AA | 4/67 |  | 1.39 (0.43-4.49) | 0.585 |  | 8/67 |  | | 0.95 (0.42-2.18) | 0.908 |  | 15/67 |  | | 1.09 (0.57-2.10) | 0.789 |  | 2/67 |  | | 0.73 (0.16-3.37) | 0.690 |
|  | GA/AA | 19/384 |  | 1.18 (0.57-2.45) | 0.661 |  | 56/384 |  | | 1.12 (0.71-1.75) | 0.628 |  | 90/384 |  | | 1.04 (0.72-1.51) | 0.832 |  | 17/384 |  | | 1.18 (0.55-2.52) | 0.666 |
| *MAP2K4* | rs3826392 |  |  |  | 0.354^c^ |  |  |  | |  | 0.110^c^ |  |  |  | |  | 0.856^c^ |  |  |  | |  | 0.132^c^ |
|  | TT | 23/439 |  | 1.00 (reference) |  |  | 55/439 |  | | 1.00 (reference) |  |  | 100/439 |  | | 1.00 (reference) |  |  | 23/439 |  | | 1.00 (reference) |  |
|  | TG | 8/225 |  | 0.65 (0.28-1.48) | 0.304 |  | 33/225 |  | | 1.21 (0.75-1.95) | 0.430 |  | 49/225 |  | | 0.92 (0.62-1.38) | 0.689 |  | 5/225 |  | | 0.42 (0.16-1.12) | 0.082 |
|  | GG | 1/25 |  | 0.78 (0.10-6.20) | 0.814 |  | 6/25 |  | | 2.46 (0.92-6.58) | 0.073 |  | 7/25 |  | | 1.51 (0.60-3.81) | 0.388 |  | 1/25 |  | | 0.73 (0.09-5.66) | 0.760 |
|  | TG/GG | 9/250 |  | 0.66 (0.30-1.46) | 0.305 |  | 39/250 |  | | 1.32 (0.84-2.07) | 0.233 |  | 56/250 |  | | 0.97 (0.66-1.43) | 0.885 |  | 6/250 |  | | 0.45 (0.18-1.12) | 0.087 |
| *MAPK9* | rs6703 |  |  |  | 0.572^c^ |  |  |  | |  | 0.881^c^ |  |  |  | |  | 0.819^c^ |  |  |  | |  | 0.883^c^ |
|  | TT | 21/463 |  | 1.00 (reference) |  |  | 65/463 |  | | 1.00 (reference) |  |  | 107/463 |  | | 1.00 (reference) |  |  | 19/463 |  | | 1.00 (reference) |  |
|  | TA | 9/201 |  | 1.10 (0.49-2.47) | 0.824 |  | 23/201 |  | | 0.77 (0.46-1.29) | 0.318 |  | 42/201 |  | | 0.86 (0.57-1.30) | 0.468 |  | 10/201 |  | | 1.28 (0.58-2.82) | 0.543 |
|  | AA | 2/25 |  | 1.68 (0.36-7.82) | 0.510 |  | 6/25 |  | | 2.14 (0.80-5.75) | 0.132 |  | 7/25 |  | | 1.31 (0.50-3.42) | 0.583 |  | 0/25 |  | | NA | 0.975 |
|  | TA/AA | 11/226 |  | 1.17 (0.55-2.50) | 0.683 |  | 29/226 |  | | 0.89 (0.55-1.43) | 0.626 |  | 49/226 |  | | 0.90 (0.61-1.34) | 0.604 |  | 10/226 |  | | 1.12 (0.51-2.45) | 0.784 |
| *MAP3K4* | rs1488 |  |  |  | 0.079^c^ |  |  |  | |  | 0.902^c^ |  |  |  | |  | 0.623^c^ |  |  |  | |  | 0.899^c^ |
|  | AA | 22/373 |  | 1.00 (reference) |  |  | 47/373 |  | | 1.00 (reference) |  |  | 82/373 |  | | 1.00 (reference) |  |  | 16/373 |  | | 1.00 (reference) |  |
|  | AG | 9/261 |  | 0.57 (0.26-1.28) | 0.172 |  | 41/261 |  | | 1.18 (0.74-1.87) | 0.491 |  | 64/261 |  | | 1.03 (0.70-1.52) | 0.879 |  | 10/261 |  | | 0.92 (0.41-2.07) | 0.841 |
|  | GG | 1/55 |  | 0.28 (0.04-2.14) | 0.220 |  | 6/55 |  | | 0.82 (0.33-2.05) | 0.672 |  | 10/55 |  | | 0.73 (0.34-1.53) | 0.340 |  | 3/55 |  | | 1.24 (0.35-4.46) | 0.738 |
|  | AG/GG | 10/316 |  | 0.52 (0.24-1.12) | 0.095 |  | 47/316 |  | | 1.11 (0.72-1.74) | 0.633 |  | 74/316 |  | | 0.97 (0.67-1.41) | 0.891 |  | 13/316 |  | | 0.98 (0.46-2.08) | 0.957 |
|  | rs678290 |  |  |  | 0.305^c^ |  |  |  | |  | 0.863^c^ |  |  |  | |  | 0.489^c^ |  |  |  | |  | 0.800^c^ |
|  | TT | 25/499 |  | 1.00 (reference) |  |  | 69/499 |  | | 1.00 (reference) |  |  | 110/499 |  | | 1.00 (reference) |  |  | 21/499 |  | | 1.00 (reference) |  |
|  | TC | 7/168 |  | 0.79 (0.33-1.90) | 0.604 |  | 23/168 |  | | 1.10 (0.65-1.84) | 0.729 |  | 43/168 |  | | 1.41 (0.92-2.15) | 0.114 |  | 8/168 |  | | 1.12 (0.48-2.60) | 0.789 |
|  | CC | 0/22 |  | NA | 0.984 |  | 2/22 |  | | 0.58 (0.13-2.60) | 0.480 |  | 3/22 |  | | 0.57 (0.16-2.04) | 0.391 |  | 0/22 |  | | NA | 0.977 |
|  | TC/CC | 7/190 |  | 0.70 (0.29-1.68) | 0.424 |  | 25/190 |  | | 1.03 (0.62-1.70) | 0.920 |  | 46/190 |  | | 1.29 (0.86-1.95) | 0.224 |  | 8/190 |  | | 1.01 (0.44-2.34) | 0.978 |
| *MAPK14* | rs3804451 |  |  |  | 0.808^c^ |  |  |  | |  | 0.192^c^ |  |  |  | |  | 0.673^c^ |  |  |  | |  | 0.849^c^ |
|  | GG | 24/492 |  | 1.00 (reference) |  |  | 62/492 |  | | 1.00 (reference) |  |  | 109/492 |  | | 1.00 (reference) |  |  | 20/492 |  | | 1.00 (reference) |  |
|  | GA | 7/179 |  | 0.82 (0.34-1.96) | 0.652 |  | 29/179 |  | | 1.46 (0.89-2.39) | 0.131 |  | 42/179 |  | | 1.12 (0.73-1.70) | 0.608 |  | 9/179 |  | | 1.15 (0.51-2.59) | 0.742 |
|  | AA | 1/18 |  | 1.27 (0.15-10.59) | 0.826 |  | 3/18 |  | | 1.19 (0.33-4.36) | 0.791 |  | 5/18 |  | | 1.03 (0.35-3.05) | 0.960 |  | 0/18 |  | | NA | 0.979 |
|  | GA/AA | 8/197 |  | 0.86 (0.37-1.97) | 0.712 |  | 32/197 |  | | 1.43 (0.89-2.30) | 0.139 |  | 47/197 |  | | 1.11 (0.74-1.66) | 0.623 |  | 9/197 |  | | 1.04 (0.46-2.35) | 0.922 |
| a Data were calculated using unconditional logistic regression, adjusted by age at diagnosis, sex, ECOG score, BMI, histological type and type of treatment regimen.. | | | | | | | | | | | | | | | | | | | | | | | |
| ^b^ Data were calculated using unconditional logistic regression, adjusted by age at diagnosis, sex, ECOG score and type of treatment regimen. | | | | | | | | | | | | | | | | | | | | | | | |
| ^c^ *P*trend: *P* value for trend tests.. | | | | | | | | | | | | | | | | | | | | | | | |
| Abbreviations: CI, confidence interval; OR, odds ratio; NA, not applicable. The results were in bold, if *P* < 0.05. | | | | | | | | | | | | | | | | | | | | | | | |
|  | | | | | | | | | | | | | | | | | | | | | | | |
|  |  |  |  |  |  |  |  |  | |  |  |  |  |  | |  |  |  |  |  | |  |  |
